# Supplementary material for: Identification of potential therapeutic targets in prostate cancer through a cross‐species approach
Source: EMBO Mol Med. 2018 Feb 5;10(3):e8274. doi: 10.15252/emmm.201708274 (PMC5840539; doi:10.15252/emmm.201708274)
Supplement: Supplementary file 1 — Appendix [file EMMM-10-e8274-s001.pdf]

Appendix

Table of Contents

- **Appendix Supplementary Figure**
- **Appendix Supplementary Methods**
- **Appendix Supplementary References**

Appendix Supplementary Figure

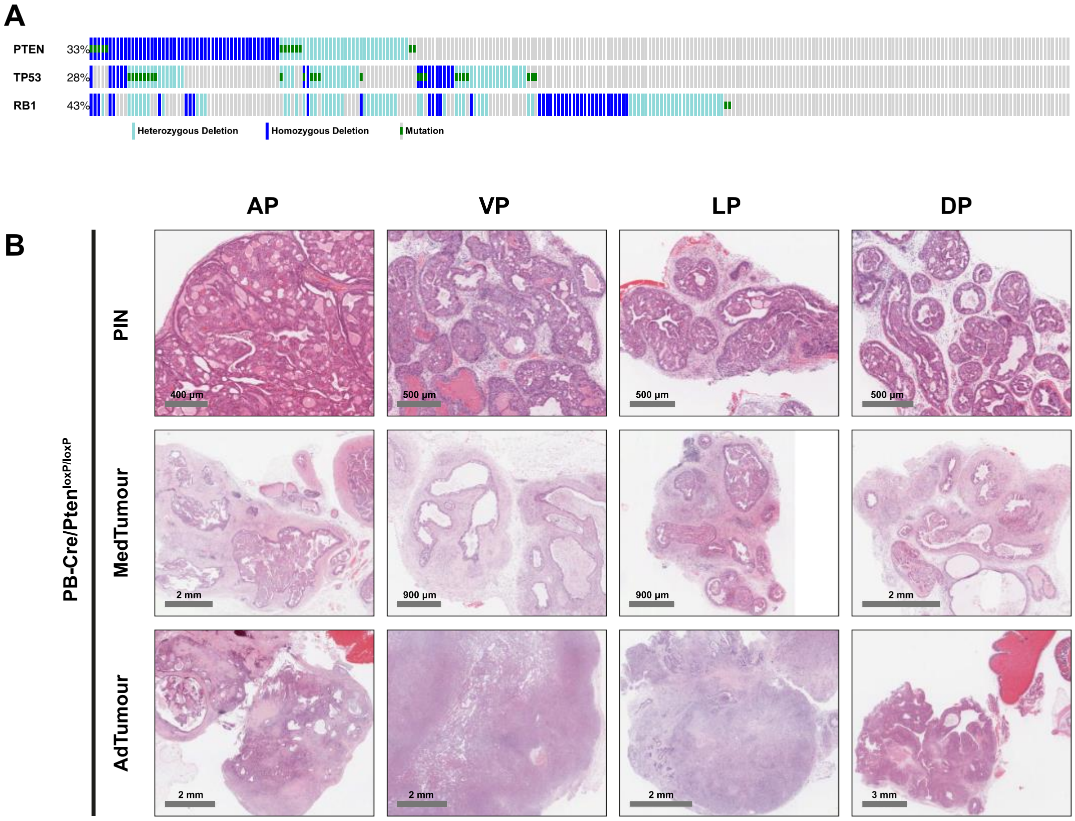

Appendix Figure S1: A: Deletions and mutations of PTEN, p53 and RB1 are frequent events in human prostate cancer. The Cancer Genome Atlas (TCGA) prostate cancer dataset was queried for heterozygous and homozygous deletions and mutations using the cBioPortal for Cancer Genomics (http://www.cbioportal.org/public-portal/)(Cerami *et al*, 2012; Gao *et al*, 2013). Data is shown for all tumours with sequencing and copy number data (n = 258). B: Representative histopathological images of the PB-Cre/Pten$^{loxP/loxP}$ mice prostatic lobes at different stages of cancer development and progression at low magnification. PIN: prostatic intraepithelial neoplasia; MedTumour: medium-stage tumour; AdTumour: advanced-stage tumour. AP: anterior prostate; VP: ventral prostate; LP: lateral prostate; DP: dorsal prostate.

Appendix Supplementary Methods

In vivo studies

Mice were maintained in the Cancer Research UK Cambridge Institute Animal Facility of the University of Cambridge and all experiments were performed in accordance with national guidelines and regulations, and with the approval of the animal care and use committee at the institution under UK Home Office project license 80/2435. The facility was specific pathogen free (SPF) and the mice were kept at 20–22 °C, in a 12-h light, 12-h dark cycle in individually ventilated cages (IVC) in groups of 5 mice when possible, with food and water ad libitum and provided with environmental enrichment. Health and welfare assessments were performed daily for all mice prior and during the studies.

Transgenic mice with the right genotype to develop prostate tumours were consolidated after weening to allow them to age together. These mice were then palpated weekly from 2 months of age to detect the emergence of tumours, whose growth was then followed up by ultrasound imaging using the high-contrast ultrasound Vevo 2100 System (50MHz, 1.5mm, 15sections). Mice were anaesthetised with isoflurane for this imaging procedure.

For xenograft studies, immunocompromised NSG male mice (Charles River, Wilmington, MA) were implanted subcutaneously with 2 million luciferase-expressing C4-2b cells in a 50:50 mixture of PBS and Phenol-red free matrigel high concentration (BD, Franklin Lakes, NJ, USA). Tumours were established for one week. Following that, mice were randomized by cage and dosed daily with either vehicle (10% DMSO in PBS) or 10 mg/kg OTS167 in 10% DMSO/90% PBS intraperitoneally, alternating daily the site of injection between the left and right sides of the mouse. Tumour volumes were measured by calliper twice weekly by a scientist not involved in the design nor analysis of the study and weekly with serial bioluminescence imaging (BLI). For BLI a sterile 15 mg/ml solution of D-luciferin potassium salt in PBS (Caliper Life Sciences, Hopkinton, MA, USA) was injected intraperitoneally to obtain a dose of 150 mg/kg. Mice were anaesthetised with isoflurane and imaged using a Xenogen IVIS imaging system. A sequence of ten images was recorded in order to capture peak luminescence for each individual tumour. Luminescence measurements were analysed using Xenogen Living Image® 3.0.

Animals were euthanized at the end of the studies by cervical dislocation and death was confirmed by verification of permanent cessation of blood circulation.

We did not perform any statistical method to choose the group size of the in vivo studies, as we did not have enough information on the variability of the model being used. For that reason, we chose to use an n=10 for each animal group, expecting that such size would provide enough power to the study to be able to detect the effects induced by the treatments.

Selection and Preparation of Mouse Prostate Samples for RNA Sequencing

To select prostate tumour samples, prostatic lobes of PB-Cre/Pten$^{loxP/loxP}$ and PB-Cre/p53$^{loxP/loxP}$Rb$^{loxP/loxP}$ mice were collected separately and sectioned into two pieces; one was snap frozen and stored at -80 °C, the other was formalin-fixed, paraffin-embedded, sectioned and stained with haematoxylin and eosin (H&E). As normal controls, prostatic lobes of Cre-negative mice were separated and the entire lobe was immersed in RNAlater (Sigma-Aldrich) prior to freezing. Prostate lesions were histopathologically classified according to recommendations from the Mouse Models of Human Cancer Consortium Prostate Pathology Committee (Ittmann *et al*, 2013; Shappell *et al*, 2004). Samples from PB-Cre/Pten$^{loxP/loxP}$ mice were divided into PIN (samples with predominantly high-grade PIN), medium-stage tumours (samples with a mixture of HG-PIN and areas of microinvasive adenocarcinoma; small areas of intracystic adenocarcinomas and intestinal metaplasia were also present) and advanced-stage tumours (samples with a mixture of HG-PIN and well-, moderate- and poorly-differentiated invasive adenocarcinoma; areas of intracystic adenocarcinomas, intestinal metaplasia and sarcomatoid differentiation were also present). Samples from PB-Cre/p53$^{loxP/loxP}$ Rb$^{loxP/loxP}$ mice were divided into PIN (glandular lobes with low-grade PIN lesions) and tumours (intra-urethral or extra-prostatic moderate- to poorly-differentiated invasive adenocarcinoma).

Based on the histopathological classification, a total of 94 samples were selected for inclusion in the study, including 20 normal prostatic lobes as well as PIN and tumours from both mouse models. Detailed information on all samples included in the RNASeq study, including strain, genotype and age of each individual animal, can be found in Table EV1.

Total RNA was extracted using the AllPrep DNA/RNA Mini Kit (Qiagen) according to the manufacturer's instructions. RNA integrity was determined using an Agilent 2100 Bioanalyzer (Agilent Technologies). Only samples with a RNA integrity number (RIN) score of ≥ 8.0 were included in the study.

Library Preparation and RNA Sequencing

Library preparation was carried out using the Illumina® TruSeq Stranded mRNA Library Prep Kit (Life Technologies) according to standard Illumina protocols. 50 bp single-end sequencing reads were generated using an Illumina HiSeq 2500 sequencing system. Sequencing reads were aligned to the human genome version hg19 or the mouse genome version mm10 using TopHat (Trapnell *et al*, 2009). Read counts were normalised and differential gene expression analysis was carried out using the DESeq workflow (Anders & Huber, 2010).

The raw and normalized data files are deposited in Gene Expression Omnibus (GEO), accession number: GSE94570 (MELK regulated genes) and GSE94574 (mouse prostate lobe gene expression).

Gene Set Enrichment Analysis

Genes in the Grasso dataset (Grasso *et al*, 2012) were ranked from most under expressed to most overexpressed in primary cancer compared to normal/benign tissue, or in metastatic samples compared to primary cancer, according to t statistic. Gene set enrichment analysis (GSEA) was performed using the GSEA Preranked tool within the GSEA software (Broad Institute, http://www.broadinstitute.org/gsea/index.jsp) (Mootha *et al*, 2003; Subramanian *et al*, 2005). The analysis was performed using 1000 permutations and the weighted enrichment statistic option.

Enrichment Analysis

To identify process networks enriched among gene sets of interest, the one-click process networks enrichment analysis tool within MetaCore™ (Thomson Reuters, Cambridge, UK) was used. The definition of various gene sets of interest is described in the respective figure legends. To compare enriched process networks between different gene sets, enrichment analysis was performed separately for each gene set, the resulting process networks were ranked according to the p-value of the enrichment, and the ranks were compared between different gene sets.

Survival Analysis

Survival analysis was carried out using a Galaxy-based (https://galaxyproject.org/) tool developed by the Cancer Research UK Cambridge Institute Bioinformatics Core Facility based on gene expression data with associated clinical follow-up from the Glinsky (Glinsky *et al*, 2004) and Taylor (Taylor *et al*, 2010) datasets. Recursive partitioning was used to determine expression level cut-offs for the gene of interest that result in the strongest association with recurrence. Associated p-values were computed, and Kaplan-Meier plots using optimal cut-offs are generated. In the case of the Taylor dataset, which includes data from both primary tumours and metastases, only primary tumours were included in the analysis.

Tissue microarray

The TMA used in this study has been previously described (Pértega-Gomes et al., 2013). Briefly, Prostate tissue was obtained from 323 patients who underwent radical prostatectomy between 1993 and 2003. The median age of patients was 64 years (46 – 74 years). A TMA was constructed using representative samples of adjacent non-neoplastic prostate tissue, PIN lesions and prostate cancer. Three tissue cores of 1 mm diameter were used for each sample.
For immunohistochemical staining of 4 µm tissue sections, the Vectastain Elite ABC Kit (Vector Laboratories, Burlingame, CA) was used as previously described (Pinheiro et al., 2008). The primary antibody directed against MELK (HPA017214, Sigma-Aldrich) was used at a dilution of 1:100.

The extent of staining was scored as 0 (0% of cells stained), 1 (< 5% of cells stained), 2 (5 – 50 % of cells stained) and 3 (> 50% of cells stained), and the intensity of staining was scored as 0 (negative), 1 (weak), 2 (moderate) and 3 (strong). To obtain the final score, the scores for extent of staining and intensity of staining were summed. Samples with a final score of 0 to 3 were considered negative, and samples with a final score of 4 to 6 were considered positive. Where discordant results were obtained for different cores derived from the same sample, the average score of the extension was added to the highest intensity score. Additionally, the subcellular localisation of the staining (cytoplasmic, nuclear or both) was noted.

Statistical analysis was performed using SPSS (version 17.0, SPSS Inc., Chicago, IL, USA). Statistical significance was tested using Pearson's chisquare ($\chi 2$) test with a significance threshold of $p < 0.05$.

Cell Lines and Culture Conditions

LNCaP, PC-3 and DU-145 cells were obtained from ATCC (Manassas, VA, USA), C4-2 and C4-2b from MD Anderson Cancer Center (Houston, TX, USA). Cells were grown at 37 °C and 5% CO2. All cell lines were maintained in RPMI 1640 (InvitrogenTM, Life Technologies, Carlsbad, CA, USA) supplemented with 10% foetal bovine serum (Invitrogen) and passaged twice per week. For passaging, cells were washed with PBS pH 7.2 (Invitrogen), detached with 0.25% Trypsin/EDTA (Invitrogen) for 3 min at 37°C, resuspended in growth medium and re-seeded.

Transient Transfection

siRNAs against MELK (MELK siRNA #1: QiagenSI02224558; MELK siRNA #2: QiagenSI02224565; MELK siRNA #3: DharmaconJ-004029-06) were transfected using Lipofectamine RNAiMAX (Invitrogen) according to the manufacturer's reverse transfection protocol. AllStars Negative Control siRNA (Qiagen SI03650318) was used as a non- targeting control. siRNAs were transfected at a final concentration of 20 nM.

Viability Assays

For viability assays using Vi-CELL, cells were seeded in 6-well plates and treated as required. All treatments were carried out in triplicate. At various time points depending on the experiment, cells in each well were harvested by trypsinisation and combined with their respective supernatant medium to recover dead cells. Cells were centrifuged at 272 X g for 5 min. The medium was aspirated, and the cell pellets were resuspended in 500 µl PBS. Viable and non-viable cells were quantified using a Vi-CELL Cell Viability Analyser (Beckman Coulter, Pasadena, CA, USA).

For MTS assays, cells were seeded in 96-well plates in 100 μl growth medium per well. Cells were allowed to attach for 24 h and then treated as required for

72 h. To prepare MTS reagent, a solution of 0.92 mg/ml phenazine methosulfate (Sigma-Aldrich) in PBS was combined with a solution of 2 mg/ml MTS reagent powder (Promega, Madison, WI, USA) in PBS at a ratio of 1:20, aliquoted and stored at -20 °C in the dark until use. 20 μl MTS reagent was added to each well, and cells were incubated at 37 ° C for 1 h in the dark. Absorbance at 490 nm was quantified using a Tecan Infinite M200 spectrophotometer (Tecan Group, Maennedorf, Switzerland).

Apoptosis Assay

Apoptotic cells were quantified based on Annexin V and PI staining. C4-2b cells were seeded in 6-well plates and treated as required; three wells were used for each treatment condition. Cells were harvested by trypsinisation, the three replicate wells were pooled, and cells were collected by centrifugation at 272 X g for 3 min. Cells were then washed twice in cold PBS and resuspended in Annexin V Binding Buffer (BioLegend, San Diego, CA, USA). Cells were counted, and the concentration of the suspension was adjusted to 1 Mio. cells/ml using the Binding Buffer. 100 μl of cell suspension were transferred to FACS tubes. 5 μl of Alexa Fluor® 647 Annexin V (Biolegend) and 10 μl of a 1 mg/ml PI solution (Sigma-Aldrich) were added. The suspension was vortexed gently and incubated for 15 min at room temperature in the dark. 400 μl Annexin V Binding Buffer were added. Alexa Fluor® 647 and PI fluorescence were quantified using a FACS Calibur (BD Biosciences). Data was analysed using FlowJo v9. Cell were gated according to side scatter to forward scatter ratio, and divided into four populations: Annexin-/PI- (live cells), Annexin+/PI- (apoptotic cells), Annexin-/PI+ and Annexin+/PI+ (dead cells).

Clonogenicity Assay

C4-2b cells were seeded at 2,000 cells/well into 6 well plates. After 48 h, cells were treated with vehicle or OTS167. The growth medium was replaced and fresh drug added twice per week. 11 days after seeding, cells were fixed with acetone-methanol 1:1 for 3 min, air-dried and stained with Giemsa (1:10 dilution in H_2O, Thermo Fisher Scientific). Colonies were quantified using a Gel Count imaging system.

Antibody array

For antibody arrays, C4-2b cells were treated with vehicle or 30 nMOTS167 for 2h. Lysates were collected and analysed using Phospho Explorer Antibody Arrays (Full Moon Biosystems, Sunnyvale, CA, USA) according to the manufacturer's instructions. Slides were scanned using a Typhoon Trio+ instrument (GE Healthcare), and spot intensities were quantified using Image Quant TL. For data analysis, means of duplicate spots were calculated, and signal from phospho-site-specific antibodies was normalised to signal from antibodies

recognising the total protein. Antibodies with high variation between duplicate spots or spatial artefacts were removed from the analysis.

Western Blot

To extract protein from tissue, 20 – 30 mg of tissue was homogenised in 350 µl ice-cold M-PER buffer (Pierce / Thermo Fisher Scientific) with protease and phosphatase inhibitor cocktails using a Precellys homogeniser and incubated for 2 h with overhead rotation at 4°C. To extract proteins from cell lines, cells were incubated with ice-cold M-PER buffer containing protease and phosphatase inhibitor cocktails for 5 min and harvested by scraping. All lysates were cleared by centrifugation at maximum speed and 4°C for 10 - 15 min in a benchtop centrifuge. Protein content was quantified using a Direct Detect Spectrometer (Millipore). Samples for Western Blot were prepared by addition of 5x Laemmli buffer and boiled for 5 min. Equal amounts of protein were loaded onto Criterion precast gels (Bio-Rad Laboratories, Hercules, CA, USA) and separated by SDS-PAGE. Proteins were transferred to nitrocellulose membranes using the iBlot™ Dry Blotting System (Invitrogen), and transfer was assessed by staining with Ponceau S (Sigma). Membranes were blocked in 5% or BSA (Sigma) in Tris-buffered saline with 0.1% Tween-20 and incubated with primary antibodies and HRP-conjugated secondary antibodies (Dako). Detection was carried out using Western Lightning ECL Pro (Perkin Elmer). The following antibodies were used (dilution used indicated in brackets): Anti-RRN3 pSer649 (ab138651, 1:1,000) and anti-β-actin (ab6276, 1:100,000), both from Abcam, Cambridge, UK; anti-stathmin pSer38 (4191, 1:1,000), anti-p90RSK pThr573 (9346, 1:1,000), anti-Bad pSer112 (9291, 1:1,000), anti-RSK1/2/3 (9355, 1:1,000), anti-Bad (9292, 1:1,000), anti-stathmin (3352, 1:1,000), anti-ACC pSer79 (3661, 1:500), all from Cell Signalling Technology, Cambridge, UK; anti-MELK (HPA017214, 1:1,000) from Sigma-Aldrich, St Louis, MO, USA; anti-RRN3 (sc-133978, 1:200), Santa Cruz Biotechnology, Dallas, TX, USA

Immunofluorescence

Cells were grown on coverslips, treated as indicated, fixed with ice-cold methanol for 6 min at -20° and permeabilised with 0.1% Tween-20 in PBS for 5 min at room temperature. Cells were then blocked with 5% BSA in PBS for 1 h at room temperature and incubated with anti-α-tubulin antibody (DM1A, Sigma) at 1:1000 dilution in blocking solution at 4°C overnight. Incubation with Alexa Fluor® 488 donkey anti-mouse antibody (Life Technologies) was performed for 1 h at room temperature at a dilution of 1:1000. Coverslips were mounted onto slides using ProLong® Gold Antifade Mountant with DAPI (Thermo Fisher Scientific). Images were obtained using a Leica SP5 confocal microscope. Leica Application Suite Advanced Fluorescence was used to export images.

Immunohistochemistry

For fixation, freshly dissected mouse tissues were placed in 4% neutral-buffered formaldehyde (Acquascience, Brambleside, UK) for 24 h and then transferred to 70 % ethanol (Sigma-Aldrich). Samples were paraffin embedded within three days of transfer to 70% ethanol.

Tissues sections were stained using a BOND-MAX Autostainer (Leica Biosystems, Nussloch, Germany). Antigen retrieval was performed at 100 °C in Bond epitope retrieval 2 diluent or Sanger diluent. Sections were incubated with primary antibodies for 15 min at room temperature and with a polymer secondary system for 8 min, and developed with diaminobenzidine. Automated haematoxylin counterstaining was performed. Slides were dehydrated, cleared and mounted using an automated CV5030 coverslipper (Leica) and scanned using the Aperio Scan Scope system (Leica).

For IHC staining of MELK in mouse tissue, paraffin - embedded sections were dewaxed in xylene and rehydrated in graded ethanol. Antigen retrieval was performed by microwaving the slides in citrate buffer (pH 6.0) for 15 min. The slides were cooled for 20 min and incubated with 1% normal donkey serum for 1 h before incubation with the primary antibody (anti-MELK, NBP1-19598, Novus Biologicals, Littleton, CO, USA) at a 1:200 dilution for 2 h at room temperature. The slides were then incubated with a 1:200 dilution secondary antibody (Goat Anti - Rabbit IgG, P 0448, Dako, Denmark) for 1 h followed. The slides were then visualised using 3,3′ - diaminobenzadine (Vector laboratories, SK - 4100) and counterstained with haematoxylin (Vector Laboratories H - 3404).

CC3 staining was quantified using the Aperio Image Scope software. For each sample a minimum of three representative images were selected for quantification. Nuclei were divided into four categories (0, 1, 2 and 3) depending on their staining intensity, and the percentage of nuclei falling into each category was computed. Nuclei with a score of 0 and 1 were considered negative, while nuclei with a score of 2 and 3 were considered positive. Results obtained from multiple images derived from the same sample were averaged.

qRT-PCR

RNA was isolated using the RNeasy Plus Mini Kit according to the manufacturer's instructions. cDNA was synthesized using the High Capacity cDNA Reverse Transcription Kit (Applied Biosystems, Foster City, CA, USA) following the manufacturer's instructions. qRT-PCR reactions were performed using on an ABI PRISM 7900 HT Sequence Detection System. Relative gene expression was calculated per the ΔΔCt method; MELK (Applied Biosystems, Mn 00487680_m1), HPRT (Applied Biosystems, Mm 03024075_m1) was used as housekeeping gene.

Data Analysis and Graphical Representation

Statistical analysis was performed in GraphPad Prism6 unless otherwise indicated. Details of statistical tests used are given in the respective figure legends. Significance levels are denoted as follows: * $p< 0.05$; ** $p < 0.01$; *** $p < 0.001$. All error bars indicate standard error of mean unless otherwise stated. Hypergeometric tests were performed in R version 3.1.1 (R Core Team, 2014) using the phyper function in the stats package. Heatmaps were generated using the gplots and R ColorBrewer packages. In case of RNASeq data, normalised counts were used as the input.

Appendix Supplementary References

Anders S & Huber W (2010) Differential expression analysis for sequence count data. *Genome Biol.* **11:** R106

Cerami E, Gao J, Dogrusoz U, Gross BE, Sumer SO, Aksoy BA, Jacobsen A, Byrne CJ, Heuer ML, Larsson E, Antipin Y, Reva B, Goldberg AP, Sander C & Schultz N (2012) The cBio cancer genomics portal: an open platform for exploring multidimensional cancer genomics data. *Cancer Discov.* **2:** 401–4

Gao J, Aksoy BA, Dogrusoz U, Dresdner G, Gross B, Sumer SO, Sun Y, Jacobsen A, Sinha R, Larsson E, Cerami E, Sander C & Schultz N (2013) Integrative analysis of complex cancer genomics and clinical profiles using the cBioPortal. *Sci. Signal.* **6:** pl1

Glinsky G V, Glinskii AB, Stephenson AJ, Hoffman RM & Gerald WL (2004) Gene expression profiling predicts clinical outcome of prostate cancer. *J. Clin. Invest.* **113:** 913–23

Grasso CS, Wu Y-M, Robinson DR, Cao X, Dhanasekaran SM, Khan AP, Quist MJ, Jing X, Lonigro RJ, Brenner JC, Asangani IA, Ateeq B, Chun SY, Siddiqui J, Sam L, Anstett M, Mehra R, Prensner JR, Palanisamy N, Ryslik GA, et al (2012) The mutational landscape of lethal castration-resistant prostate cancer. *Nature* **487:** 239–43

Ittmann M, Huang J, Radaelli E, Martin P, Signoretti S, Sullivan R, Simons BW, Ward JM, Robinson BD, Chu GC, Loda M, Thomas G, Borowsky A & Cardiff RD (2013) Animal models of human prostate cancer: The consensus report of the new york meeting of the mouse models of human cancers consortium prostate pathology committee. *Cancer Res.* **73:** 2718–2736

Mootha VK, Lindgren CM, Eriksson K-F, Subramanian A, Sihag S, Lehar J, Puigserver P, Carlsson E, Ridderstråle M, Laurila E, Houstis N, Daly MJ, Patterson N, Mesirov JP, Golub TR, Tamayo P, Spiegelman B, Lander ES, Hirschhorn JN, Altshuler D, et al (2003) PGC-1alpha-responsive genes involved in oxidative phosphorylation are coordinately downregulated in human diabetes. *Nat. Genet.* **34:** 267–73

R Core Team (2014) R: A language and environment for statistical computing. *R Found. Stat. Comput.*

Shappell SB, Thomas G V, Roberts RL, Herbert R, Ittmann MM, Rubin MA, Humphrey PA, Sundberg JP, Rozengurt N, Barrios R, Ward JM & Cardiff RD (2004) Prostate pathology of genetically engineered mice: definitions and classification. The consensus report from the Bar Harbor meeting of the Mouse Models of Human Cancer Consortium Prostate Pathology Committee. *Cancer Res.* **64:** 2270–305

Subramanian A, Tamayo P, Mootha VK, Mukherjee S, Ebert BL, Gillette MA, Paulovich A, Pomeroy SL, Golub TR, Lander ES & Mesirov JP (2005) Gene set enrichment analysis: a knowledge-based approach for interpreting genome-wide expression profiles. *Proc. Natl. Acad. Sci. U. S. A.* **102:** 15545–50

Taylor BS, Schultz N, Hieronymus H, Gopalan A, Xiao Y, Carver BS, Arora VK, Kaushik P, Cerami E, Reva B, Antipin Y, Mitsiades N, Landers T, Dolgalev I, Major JE, Wilson M, Socci ND, Lash AE, Heguy A, Eastham JA, et al (2010) Integrative genomic profiling of human prostate cancer. *Cancer Cell* **18:** 11–22

Trapnell C, Pachter L & Salzberg SL (2009) TopHat: discovering splice junctions with RNA-Seq. *Bioinformatics* **25:** 1105–11
