# Supplementary material for: Identification of potential therapeutic targets in prostate cancer through a cross‐species approach
Source: EMBO Mol Med. 2018 Feb 5;10(3):e8274. doi: 10.15252/emmm.201708274 (PMC5840539; doi:10.15252/emmm.201708274)
Supplement: Supplementary file 2 — Expanded View Figures PDF [file EMMM-10-e8274-s002.pdf]

Expanded View Figures

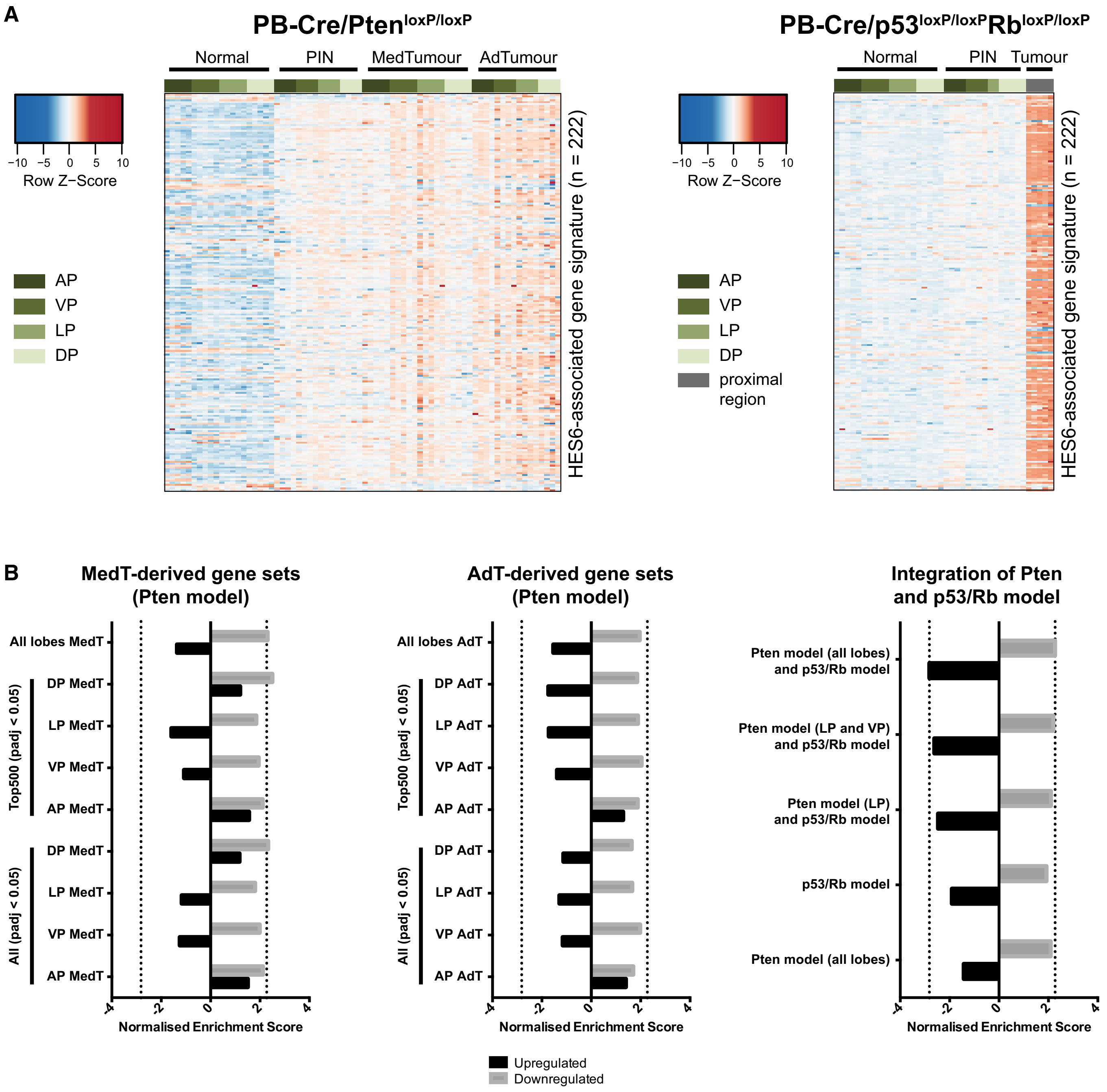

Figure EV1. Similarities between human and murine prostate cancer facilitate identification of potential therapeutic targets.

A Expression patterns of the Hes6 signature (Ramos-Montoya *et al*, 2014) mouse homologous genes in prostate tumours arising in the PB-Cre/Pten$^{loxP/loxP}$ and PB-Cre/p53$^{loxP/loxP}$Rb$^{loxP/loxP}$ models.

B Gene set enrichment analysis was used to compare various subsets of genes altered in mouse prostate cancer to the Grasso dataset of human prostate cancer (Grasso *et al*, 2012). Dotted lines indicate the NES obtained using the gene set “Pten model (all lobes) and p53/Rb model”. A significance cut-off of P_{adj} < 0.05 was used for all gene sets. Normalised enrichment scores < 0: gene set enriched in human prostate tumours; normalised enrichment score > 0: gene set enriched in benign human prostate tissue. Due to the large number of DEGs in the p53/pRb model, only the top 500 significantly upregulated and downregulated genes each were used for this gene set.

© 2018 The Authors

Figure EV2. MELK is overexpressed in human prostate cancer and associated with an aggressive phenotype.

A Expression in the Cambridge dataset (Ross-Adams *et al*, 2015) of the ten potential therapeutic targets identified by cross-species analysis. Asterisks indicate statistically significant ($P < 0.05$) differences in expression between tumour vs. benign or CRPC vs. tumour, as applicable.

B The ten potential therapeutic targets are frequently associated with poor outcome. Recursive partitioning was used to determine whether potential target genes are associated with relapse in the Taylor (Taylor *et al*, 2010), Glinsky (Glinsky *et al*, 2004), Cambridge and Stockholm datasets (Ross-Adams *et al*, 2015). In cases where significant associations were found, directionality is indicated as coloured boxes.

C Kaplan–Meier plots showing that the high expression of MELK is associated with shorter time to relapse in the Stockholm (Ross-Adams *et al*, 2015), Glinsky (Glinsky *et al*, 2004) and Taylor (Taylor *et al*, 2010) datasets. Cut-offs for MELK expression levels were determined by recursive partitioning. Statistical significance was assessed by Log-rank test.

D Expression of MELK in the Stockholm dataset in the prostate cancer iClusters identified in Ross-Adams *et al* (2015). Horizontal line indicates median; box limits correspond to 75th and 25th percentiles; whiskers correspond to 95th and 5th percentile.

E Expression of MELK across multiple prostate cancer datasets according to the Oncomine database. Colours indicate whether MELK is among the 1, 5, 10 or 25% most upregulated genes in a given dataset. Numbers correspond to the following datasets: (1) Arredouani *et al* (2009), (2) Lapointe *et al* (2004), (3) LaTulippe *et al* (2002), (4) Liu *et al* (2006), (5) Luo *et al* (2002), (6) Magee *et al* (2001), (7) Singh *et al* (2002), (8) Taylor *et al* (2010), (9) Vanaja *et al* (2003), (10) Varambally *et al* (2005), (11) Wallace *et al* (2008), (12) Welsh *et al* (2001), (13) Yu *et al* (2004).

Source data are available online for this figure.

 © 2018 The Authors

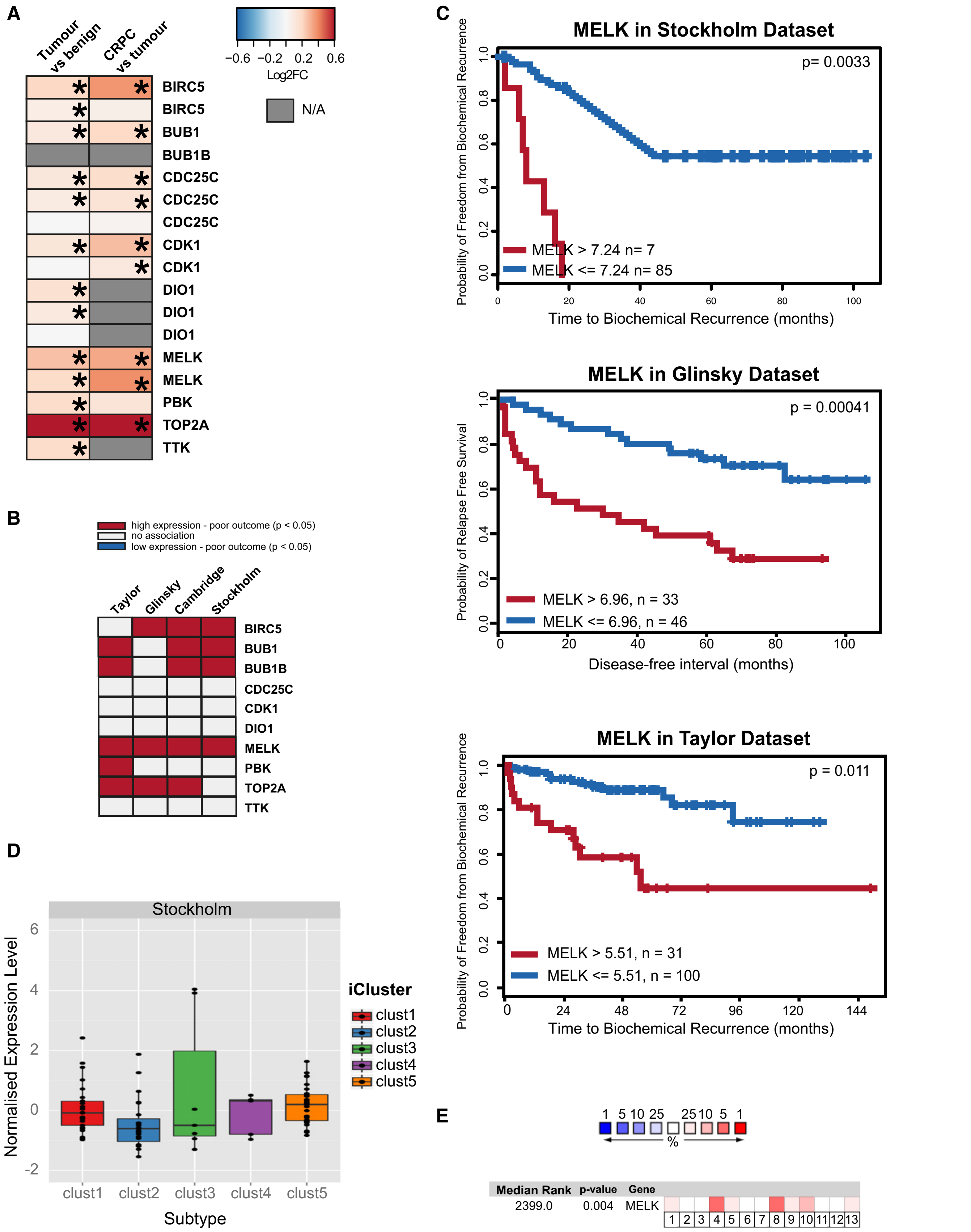
A
Tumour vs benign
CRPC vs tumour
−0.6 −0.2 0.2 0.6
Log2FC
N/A
BIRC5
BIRC5
BUB1
BUB1B
CDC25C
CDC25C
CDC25C
CDK1
CDK1
DIO1
DIO1
DIO1
MELK
MELK
PBK
TOP2A
TTK
B
high expression - poor outcome (p < 0.05)
no association
low expression - poor outcome (p < 0.05)
Taylor
Glinsky
Cambridge
Stockholm
BIRC5
BUB1
BUB1B
CDC25C
CDK1
DIO1
MELK
PBK
TOP2A
TTK
C
MELK in Stockholm Dataset
p= 0.0033
MELK > 7.24 n= 7
MELK <= 7.24 n= 85
Probability of Freedom from Biochemical Recurrence
Time to Biochemical Recurrence (months)
MELK in Glinsky Dataset
p = 0.00041
MELK > 6.96, n = 33
MELK <= 6.96, n = 46
Probability of Relapse Free Survival
Disease-free interval (months)
MELK in Taylor Dataset
p = 0.011
MELK > 5.51, n = 31
MELK <= 5.51, n = 100
Probability of Freedom from Biochemical Recurrence
Time to Biochemical Recurrence (months)
D
Stockholm
Normalised Expression Level
clust1
clust2
clust3
clust4
clust5
Subtype
iCluster
E
1 5 10 25 25 10 5 1
%
Median Rank p-value Gene
2399.0 0.004 MELK
1 2 3 4 5 6 7 8 9 10 11 12 13

Figure EV2.

Figure EV3. MELK is overexpressed in prostate tumours of PB-Cre/Pten$^{loxP/loxP}$ and PB-Cre/p53$^{loxP/loxP}$Rb$^{loxP/loxP}$ mice.

A mRNA expression levels of MELK in normal prostate, PIN and tumours of PB-Cre/Pten$^{loxP/loxP}$ and PB-Cre/p53$^{loxP/loxP}$Rb$^{loxP/loxP}$ mice as quantified by qRT–PCR. n = 2 per lobe and tumour stage, except PTEN MedTumour DP (n = 3).

B Immunohistochemical staining for MELK in normal anterior (I), ventral (II), lateral (III) and dorsal (IV) prostate showing low expression. Scale bars correspond to 200 μm.

C Immunohistochemical staining for MELK in PIN (I), well-differentiated to moderately differentiated medium-stage adenocarcinoma (II) and poorly differentiated advanced adenocarcinoma in PB-Cre/Pten$^{loxP/loxP}$ mice showing increased expression of MELK. Scale bars correspond to 200 μm.

D Immunohistochemical staining for MELK in liver with metastases (I and Ia) and intra-urethra/urethral primary prostate tumour (I and Ib), and anterior prostate PIN (II) of PB-Cre/p53$^{loxP/loxP}$Rb$^{loxP/loxP}$ mouse showing high MELK expression in PIN, primary tumour and metastases. L: liver, M: metastasis, T: primary tumour. Scale bars correspond to 3 mm (I) and 200 μm (Ia, Ib and II).

Source data are available online for this figure.

© 2018 The Authors

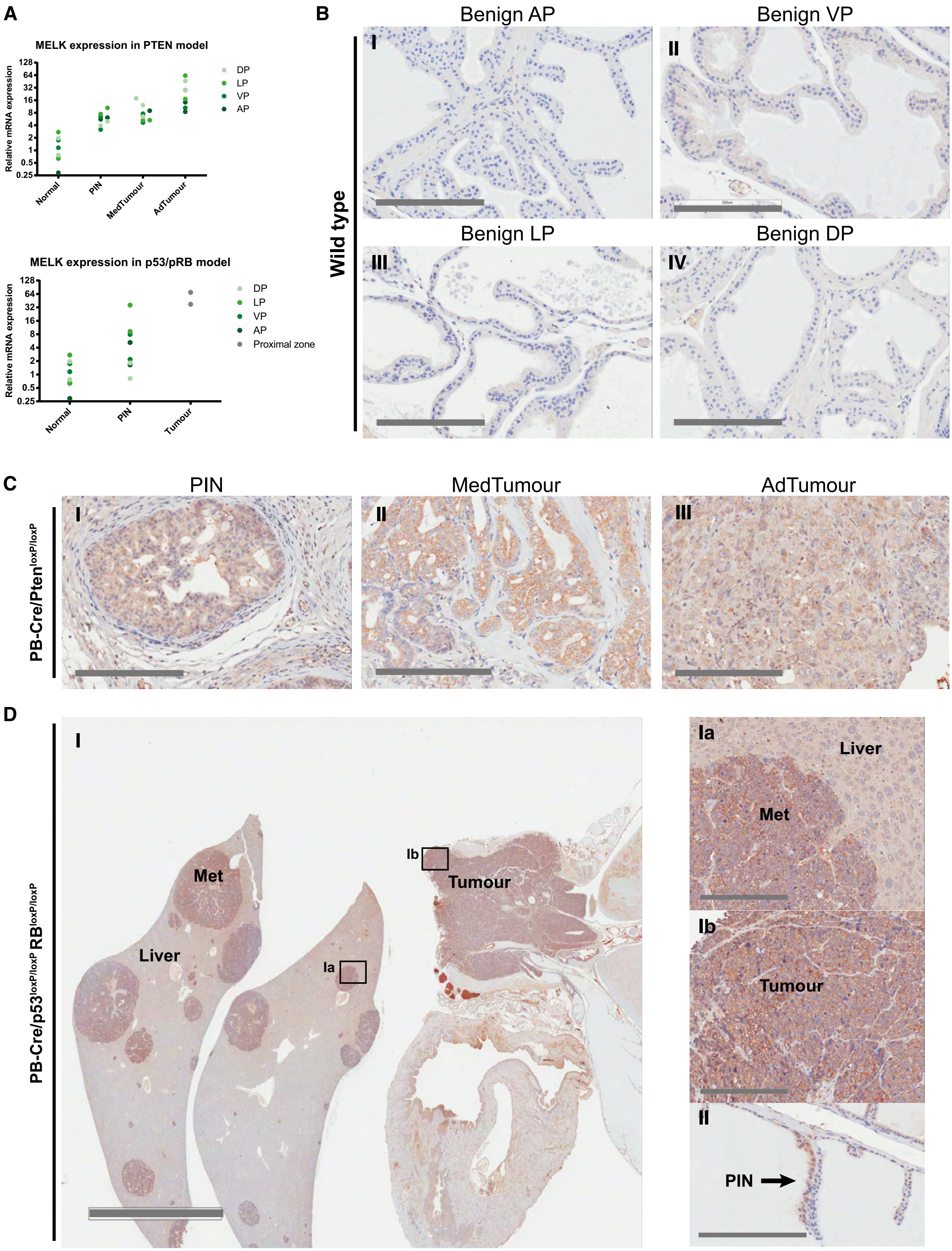
A
MELK expression in PTEN model
Relative mRNA expression
DP
LP
VP
AP
Normal
PIN
MedTumour
AdTumour
MELK expression in p53/pRB model
Proximal zone
Tumour
B
Benign AP
Benign VP
Benign LP
Benign DP
Wild type
C
PIN
MedTumour
AdTumour
PB-Cre/Pten loxP/loxP
D
PB-Cre/p53 loxP/loxP RB loxP/loxP
Met
Liver
Tumour
Ia
Ib
II
PIN

Figure EV3.

Figure EV4. Abrogation of MELK activity reduces prostate cancer cell proliferation.

A Effect of OTS167 on MELK targets. C4-2b cells were treated with 15, 30 or 60 nM OTS167 for 1 and 24 h. Phosphorylation of ACC at Ser-79 and MELK protein levels were determined by Western blot analysis. β-Actin was used as a loading control.

B Validation of MELK knock-down by three siRNAs. C4-2b cells were transfected with siRNAs, and after 48 h, MELK levels were determined by Western blot analysis. β-Actin was used as a loading control.

C Venn diagrams showing the overlaps between genes altered following silencing or inhibition of MELK. C4-2b cells were transfected with siRNAs directed against MELK for 72 h, or treated with 30 nM OTS167 for 8 and 24 h, and subjected to RNA sequencing (n = 4). Differentially expressed genes were identified compared to samples transfected with control siRNA or treated with vehicle, respectively ($P_{adj} < 0.05$).

D Expression of MELK in Hes6-overexpressing LNCaP xenografts (Ramos-Montoya *et al*, 2014).

E Effect of silencing of MELK on proliferation of prostate cancer cells. LNCaP cells were transfected with siRNAs directed against MELK or a non-targeting control, and viable cells were counted after 4 and 7 days. n = 4. Statistical significance was assessed by randomised blocks ANOVA (significance threshold of 0.05) followed by Holm–Sidak's multiple comparisons test.

F Effect of OTS167 on proliferation of prostate cancer cells. LNCaP cells were treated with vehicle or OTS167 at varying concentrations, and viable cells were counted after 2 and 5 days. n = 3. Statistical significance was assessed by randomised blocks ANOVA (significance threshold of 0.05) followed by Holm–Sidak's multiple comparisons test.

G OTS167 reduces proliferation of five prostate cancer cell lines and one non-transformed prostate cell line at nanomolar concentrations. Cells were treated with vehicle or OTS167 in concentrations ranging from 60 pM to 4.1 μM. After 72 h, viability was quantified by MTS assay and IC50 values were calculated. n = 2, with six technical replicates per biological replicate.

Source data are available online for this figure.

© 2018 The Authors

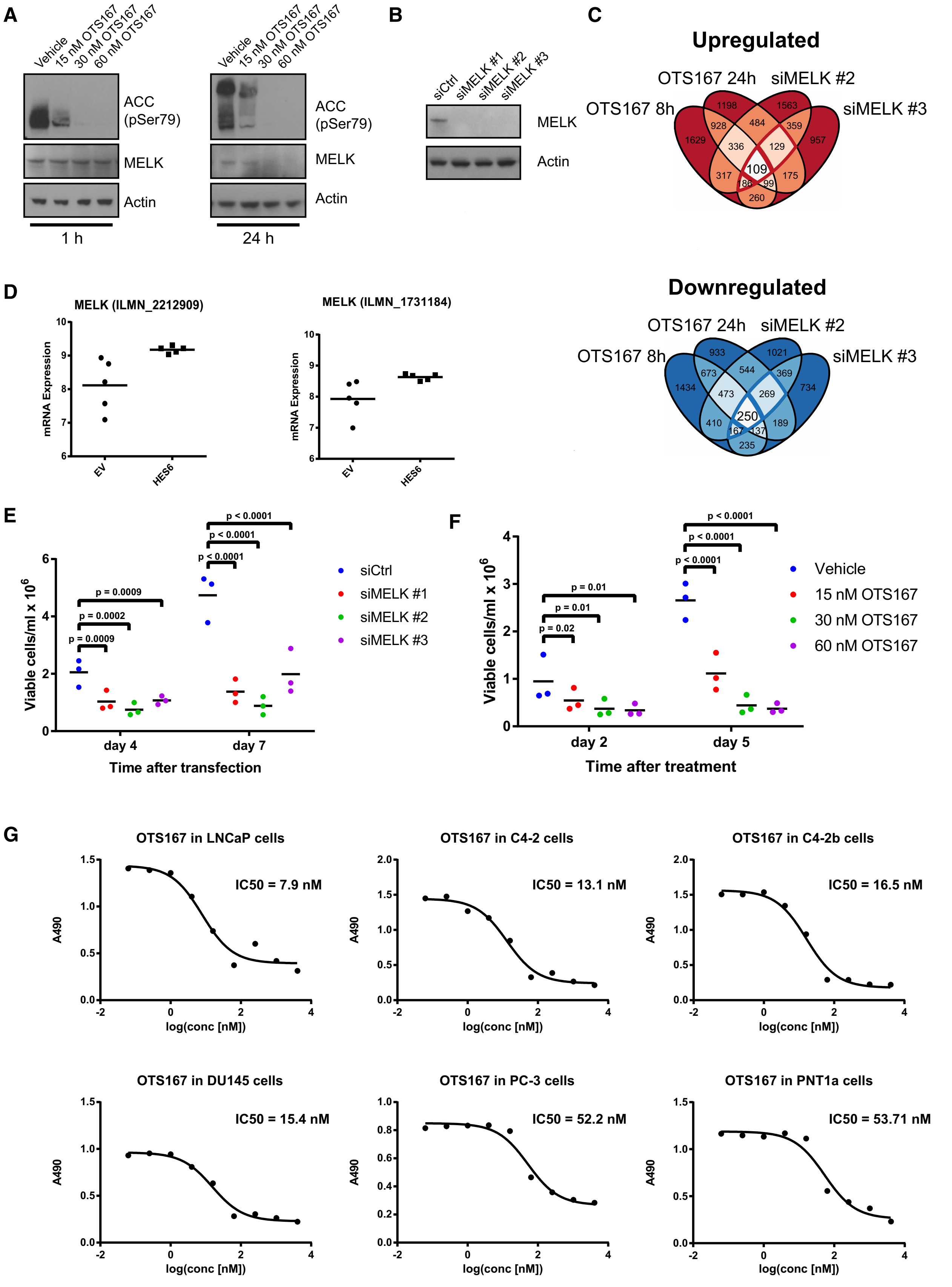
A
Vehicle
15 nM OTS167
30 nM OTS167
60 nM OTS167
ACC (pSer79)
MELK
Actin
1 h
24 h
B
siCtrl
siMELK #1
siMELK #2
siMELK #3
MELK
Actin
C
Upregulated
OTS167 24h
siMELK #2
OTS167 8h
siMELK #3
Downregulated
D
MELK (ILMN_2212909)
MELK (ILMN_1731184)
mRNA Expression
EV
HES6
E
Viable cells/ml x 10^6
p = 0.0009
p = 0.0002
p < 0.0001
siCtrl
siMELK #1
siMELK #2
siMELK #3
day 4
day 7
Time after transfection
F
p = 0.01
p = 0.02
p < 0.0001
Vehicle
15 nM OTS167
30 nM OTS167
60 nM OTS167
day 2
day 5
Time after treatment
G
OTS167 in LNCaP cells
IC50 = 7.9 nM
OTS167 in C4-2 cells
IC50 = 13.1 nM
OTS167 in C4-2b cells
IC50 = 16.5 nM
OTS167 in DU145 cells
IC50 = 15.4 nM
OTS167 in PC-3 cells
IC50 = 52.2 nM
OTS167 in PNT1a cells
IC50 = 53.71 nM
A490
log(conc [nM])

Figure EV4.

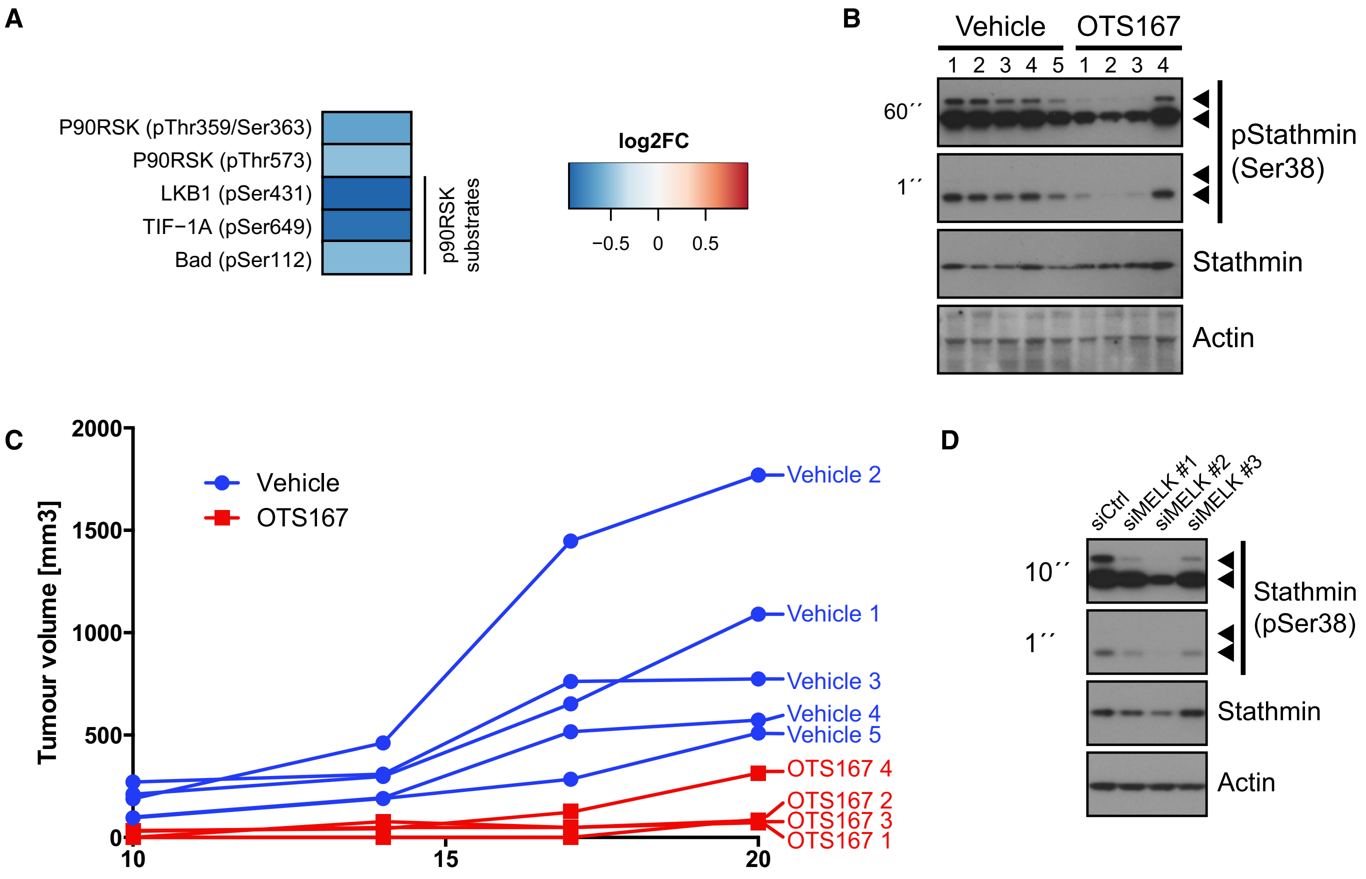

Figure EV5. Identification and validation of downstream pathways regulated by MELK.

A Phosphorylation sites on p90RSK and its substrates affected by OTS167. C4-2b cells were treated with vehicle or 30 nM OTS167 for 2 h, and phosphoproteins were analysed using a Phospho Explorer Antibody Array. Signals for each phosphorylation site were normalised to its corresponding total protein. Results for all phosphorylation sites on p90RSK and its known target sites that were analysed and exhibited a log2FC of 0.5 or greater are shown.

B OTS167 reduces phosphorylation of stathmin *in vivo*. Protein was extracted from xenograft tissue of vehicle or OTS167-treated mice, and total and phosphorylated levels of stathmin were determined by Western blot. Due to the extremely small size and high degree of cell death in OTS167-treated xenograft tumours, only four samples in the drug-treated group yielded lysates of sufficient quality.

C Individual growth curves of xenograft tumours as determined by calliper measurements are shown. Growth curves corresponding to samples in (D) are shown in brighter colours and individually labelled.

D Silencing of MELK reduces phosphorylation of stathmin at Ser-38. C4-2b cells were transfected with siRNAs directed against MELK for 72 h, and levels of total and phosphorylated stathmin were determined by Western blot analysis. β-Actin was used as a loading control.

Source data are available online for this figure.

© 2018 The Authors
