# Supplementary material for: Identification of potential therapeutic targets in prostate cancer through a cross‐species approach
Source: EMBO Mol Med. 2018 Feb 5;10(3):e8274. doi: 10.15252/emmm.201708274 (PMC5840539; doi:10.15252/emmm.201708274)
Supplement: Supplementary file 3 — Table EV1 [file EMMM-10-e8274-s003.docx]

Table EV1. Prostate samples from genetically engineered mice used for RNA Sequencing.

| Sample number | Genotype | Strain | Lobe | Animal ID | Age (months) | RNA Integrity | RNA Later*^TM^* | Prostate Histopathology |
| --- | --- | --- | --- | --- | --- | --- | --- | --- |
| 1 | *Pten^flox/flox^* | FVB | AP | 664 | 6 | 8.9 | * | Normal |
| 2 | *Pten^flox/flox^* | FVB | AP | 1058 | 6 | 8.0 | * |  |
| 3 | *Pten^flox/flox^* | FVB | AP | 1059 | 6 | 8.0 | * |  |
| 4 | *Pten^flox/flox^* | FVB | AP | 1205 | 6 | 8.3 | * |  |
| 5 | *Pten^flox/flox^* | FVB | AP | 1303 | 6 | 8.0 | * |  |
| 6 | *Pten^flox/flox^* | FVB | VP | 664 | 6 | 9.5 | * |  |
| 7 | *Pten^flox/flox^* | FVB | VP | 665 | 6 | 9.9 | * |  |
| 8 | *Pten^flox/flox^* | FVB | VP | 977 | 6 | 9.8 | * |  |
| 9 | *Pten^flox/flox^* | FVB | VP | 978 | 6 | 10 | * |  |
| 10 | *Pten^flox/flox^* | FVB | VP | 1336 | 6 | 10 | * |  |
| 11 | *Pten^flox/flox^* | FVB | LP | 977 | 6 | 9 | * |  |
| 12 | *Pten^flox/flox^* | FVB | LP | 1058 | 6 | 9.8 | * |  |
| 13 | *Pten^flox/flox^* | FVB | LP | 1205 | 6 | 9 | * |  |
| 14 | *Pten^flox/flox^* | FVB | LP | 1303 | 6 | 9.7 | * |  |
| 15 | *Pten^flox/flox^* | FVB | LP | 1336 | 6 | 10 | * |  |
| 16 | *Pten^flox/flox^* | FVB | DP | 1056a | 6 | 9 | * |  |
| 17 | *Pten^flox/flox^* | FVB | DP | 977 | 6 | 8.7 | * |  |
| 18 | *Pten^flox/flox^* | FVB | DP | 1056b | 6 | 9.2 | * |  |
| 19 | *Pten^flox/flox^* | FVB | DP | 1205 | 6 | 8.6 | * |  |
| 20 | *Pten^flox/flox^* | FVB | DP | 1303 | 6 | 8.5 | * |  |
| 21 | *ARR_2_PB-Cre;Trp53^flox/flox^Rb1^flox/flox^* | FVB | AP | 1076 | 7 | 8 | * | LG-PIN and HG-PIN |
| 22 | *ARR_2_PB-Cre; Trp53^flox/flox^Rb1^flox/flox^* | FVB | AP | 1096 | 7 | 8.2 | * |  |
| 23 | *ARR_2_PB-Cre; Trp53^flox/flox^Rb1^flox/flox^* | FVB | AP | 1122 | 7 | 8.1 | * |  |
| 24 | *ARR_2_PB-Cre; Trp53^flox/flox^Rb1^flox/flox^* | FVB | AP | 1127 | 7 | 8.4 | * |  |
| 25 | *ARR_2_PB-Cre; Trp53^flox/flox^Rb1^flox/flox^* | FVB | VP | 1076 | 7 | 9.7 | * |  |
| 26 | *ARR_2_PB-Cre; Trp53^flox/flox^Rb1^flox/flox^* | FVB | VP | 1078 | 7 | 9.8 | * |  |
| 27 | *ARR_2_PB-Cre; Trp53^flox/flox^Rb1^flox/flox^* | FVB | VP | 1122 | 7 | 9.9 | * |  |
| 28 | *ARR_2_PB-Cre;Trp53^flox/flox^Rb1^flox/flox^* | FVB | VP | 1128 | 7 | 10 | * |  |
| 29 | *ARR_2_PB-Cre; Trp53^flox/flox^Rb1^flox/flox^* | FVB | LP | 1089 | 7 | 9 | * |  |
| 30 | *ARR_2_PB-Cre; Trp53^flox/flox^Rb1^flox/flox^* | FVB | LP | 1096 | 7 | 9.8 | * |  |
| 31 | *ARR_2_PB-Cre; Trp53^flox/flox^Rb1^flox/flox^* | FVB | LP | 1122 | 7 | 9.8 | * |  |
| 32 | *ARR_2_PB-Cre; Trp53^flox/flox^Rb1^flox/flox^* | FVB | LP | 1128 | 7 | 9.5 | * |  |
| 33 | *ARR_2_PB-Cre; Trp53^flox/flox^Rb1^flox/flox^* | FVB | DP | 1089 | 7 | 9.3 | * |  |
| 34 | *ARR_2_PB-Cre; Trp53^flox/flox^Rb1^flox/flox^* | FVB | DP | 1096 | 7 | 10 | * |  |
| 35 | *ARR_2_PB-Cre;Trp53^flox/flox^Rb1^flox/flox^* | FVB | DP | 1112 | 7 | 9.7 | * |  |
| 36 | *ARR_2_PB-Cre;Trp53^flox/flox^Rb1^flox/flox^* | FVB | DP | 1127 | 7 | 9.9 | * |  |
| 37 | *ARR_2_PB-Cre; Trp53^flox/flox^Rb1^flox/flox^* | FVB | DP | 1128 | 7 | 10 | * |  |
| 38 | *ARR_2_PB-Cre; Trp53^flox/flox^Rb1^flox/flox^* | FVB | DP | 816 | 6 | 10 |  | Moderate and poorly differentiated adenocarcinoma |
| 39 | *ARR_2_PB-Cre; Trp53^flox/flox^Rb1^flox/flox^* | FVB | DP | 883 | 6.5 | 10 |  |  |
| 40 | *ARR_2_PB-Cre; Trp53^flox/flox^Rb1^flox/flox^* | FVB | DP | 1880 | 7 | 9.9 |  |  |
| 41 | *ARR_2_PB-Cre; Trp53^flox/flox^Rb1^flox/flox^* | FVB | DP | 57337 | 6.5 | 8.6 |  |  |
| 42 | *ARR_2_PB-Cre; Trp53^flox/flox^Rb1^flox/flox^* | FVB | DP | 57340 | 6.5 | 10 |  |  |
| 43 | *ARR_2_PB-Cre;Pten^flox/flox^* | FVB | AP | 1457 | 5 | 9.2 |  | HG-PIN |
| 44 | *ARR_2_PB-Cre;Pten^flox/flox^* | FVB | AP | 49 | 4.5 | 8.5 |  |  |
| 45 | *ARR_2_PB-Cre;Pten^flox/flox^* | FVB | AP | 12 | 4.7 | 10 |  |  |
| 46 | *ARR_2_PB-Cre;Pten^flox/flox^* | FVB | AP | 1170 | 6.5 | 8.7 |  |  |
| 47 | *ARR_2_PB-Cre;Pten^flox/flox^* | FVB | VP | 562 | 5 | 8.9 |  |  |
| 48 | *ARR_2_PB-Cre;Pten^flox/flox^* | FVB | VP | 1063 | 4.5 | 9.9 |  |  |
| 49 | *ARR_2_PB-Cre;Pten^flox/flox^* | FVB | VP | 1065 | 4.7 | 9.7 |  |  |
| 50 | *ARR_2_PB-Cre;Pten^flox/flox^* | FVB | VP | 1170 | 6.5 | 9.4 |  |  |
| 51 | *ARR_2_PB-Cre;Pten^flox/flox^* | FVB | LP | 1063 | 4.5 | 9.3 |  |  |
| 52 | *ARR_2_PB-Cre;Pten^flox/flox^* | FVB | LP | 1064 | 4.7 | 9.8 |  |  |
| 53 | *ARR_2_PB-Cre;Pten^flox/flox^* | FVB | LP | 1065 | 4.7 | 9 |  |  |
| 54 | *ARR_2_PB-Cre;Pten^flox/flox^* | FVB | LP | 1170 | 6,5 | 9.4 |  |  |
| 55 | *ARR_2_PB-Cre;Pten^flox/flox^* | FVB | DP | 562 | 5 | 8.6 |  |  |
| 56 | *ARR_2_PB-Cre;Pten^flox/flox^* | FVB | DP | 1064 | 4.7 | 8.6 |  |  |
| 57 | *ARR_2_PB-Cre;Pten^flox/flox^* | FVB | DP | 1065 | 4.7 | 8.7 |  |  |
| 58 | *ARR_2_PB-Cre;Pten^flox/flox^* | FVB | DP | 1170 | 6.5 | 9.1 |  |  |
| 59 | *ARR_2_PB-Cre;Pten^flox/flox^* | FVB | AP | 36848 | 13.5 | 8.2 |  | HG-PIN, desmoplatic reaction and microinvasive adenocarcinoma |
| 60 | *ARR_2_PB-Cre;Pten^flox/flox^* | FVB | AP | 37716 | 13.3 | 8.5 |  |  |
| 61 | *ARR_2_PB-Cre;Pten^flox/flox^* | FVB | AP | 39470 | 12 | 9.2 |  |  |
| 62 | *ARR_2_PB-Cre;Pten^flox/flox^* | FVB | AP | 40707 | 12.3 | 8 |  |  |
| 63 | *ARR_2_PB-Cre;Pten^flox/flox^* | FVB | AP | 41871 | 12 | 8 |  |  |
| 64 | *ARR_2_PB-Cre;Pten^flox/flox^* | FVB | VP | 39740 | 12 | 8.7 |  |  |
| 65 | *ARR_2_PB-Cre;Pten^flox/flox^* | FVB | VP | 43668 | 11.3 | 9.5 |  |  |
| 66 | *ARR_2_PB-Cre;Pten^flox/flox^* | FVB | VP | 176 | 10.5 | 9.8 |  |  |
| 67 | *ARR_2_PB-Cre;Pten^flox/flox^* | FVB | VP | 1787 | 12.2 | 9.7 |  |  |
| 68 | *ARR_2_PB-Cre;Pten^flox/flox^* | FVB | VP | 43578 | 11.4 | 10 |  |  |
| 69 | *ARR_2_PB-Cre;Pten^flox/flox^* | FVB | LP | 15620 | 20 | 9.8 |  |  |
| 70 | *ARR_2_PB-Cre;Pten^flox/flox^* | FVB | LP | 41871 | 12 | 9.2 |  |  |
| 71 | *ARR_2_PB-Cre;Pten^flox/flox^* | FVB | LP | 42331 | 11.3 | 10 |  |  |
| 72 | *ARR_2_PB-Cre;Pten^flox/flox^* | FVB | LP | 1665 | 12.3 | 9.5 |  |  |
| 73 | *ARR_2_PB-Cre;Pten^flox/flox^* | FVB | LP | 1787 | 12.2 | 9.1 |  |  |
| 74 | *ARR_2_PB-Cre;Pten^flox/flox^* | FVB | DP | 39470 | 12 | 9.6 |  |  |
| 75 | *ARR_2_PB-Cre;Pten^flox/flox^* | FVB | DP | 40707 | 12.3 | 9.6 |  |  |
| 76 | *ARR_2_PB-Cre;Pten^flox/flox^* | FVB | DP | 41871 | 12 | 9.7 |  |  |
| 77 | *ARR_2_PB-Cre;Pten^flox/flox^* | FVB | DP | 1787 | 12.2 | 8.1 |  |  |
| 78 | *ARR_2_PB-Cre;Pten^flox/flox^* | FVB | DP | 44251 | 13 | 9.2 |  |  |
| 79 | *ARR_2_PB-Cre;Pten^flox/flox^* | FVB | AP | 22157 | 17.7 | 8.5 |  | HG-PIN, desmoplatic reaction, microinvasive adenocarcinoma, well, moderate and poorly differentiated adenocarcinoma, |
| 80 | *ARR_2_PB-Cre;Pten^flox/flox^* | FVB | AP | 23422 | 17.4 | 9.3 |  |  |
| 81 | *ARR_2_PB-Cre;Pten^flox/flox^* | FVB | AP | 37718 | 13.3 | 8.8 |  |  |
| 82 | *ARR_2_PB-Cre;Pten^flox/flox^* | FVB | AP | 41229 | 12.1 | 8.8 |  |  |
| 83 | *ARR_2_PB-Cre;Pten^flox/flox^* | FVB | VP | 6 | 13.7 | 10 | * |  |
| 84 | *ARR_2_PB-Cre;Pten^flox/flox^* | FVB | VP | 71 | 9 | 8 |  |  |
| 85 | *ARR_2_PB-Cre;Pten^flox/flox^* | FVB | VP | 23422 | 17.4 | 8.8 |  |  |
| 86 | *ARR_2_PB-Cre;Pten^flox/flox^* | FVB | VP | 38123 | 13 | 9.4 |  |  |
| 87 | *ARR_2_PB-Cre;Pten^flox/flox^* | FVB | LP | 129 | 11.5 | 10 | * |  |
| 88 | *ARR_2_PB-Cre;Pten^flox/flox^* | FVB | LP | 37337 | 13.5 | 8.5 |  |  |
| 89 | *ARR_2_PB-Cre;Pten^flox/flox^* | FVB | LP | 41299 | 12.1 | 9.1 |  |  |
| 90 | *ARR_2_PB-Cre;Pten^flox/flox^* | FVB | LP | 42101 | 12 | 8.3 |  |  |
| 91 | *ARR_2_PB-Cre;Pten^flox/flox^* | FVB | DP | 52 | 12.6 | 10 | * |  |
| 92 | *ARR_2_PB-Cre;Pten^flox/flox^* | FVB | DP | 20994 | 18.2 | 8.4 |  |  |
| 93 | *ARR_2_PB-Cre;Pten^flox/flox^* | FVB | DP | 22157 | 17.7 | 8.5 |  |  |
| 94 | *ARR_2_PB-Cre;Pten^flox/flox^* | FVB | DP | 13 | 12.6 | 8.4 |  |  |
