# Supplementary material for: Identification of potential therapeutic targets in prostate cancer through a cross‐species approach
Source: EMBO Mol Med. 2018 Feb 5;10(3):e8274. doi: 10.15252/emmm.201708274 (PMC5840539; doi:10.15252/emmm.201708274)
Supplement: Supplementary file 5 — Source Data for Expanded View [file EMMM-10-e8274-s011.zip › SourceDataForFigureEV4/EMM-2017-08274_SourceDataForFigureEV4.pdf]

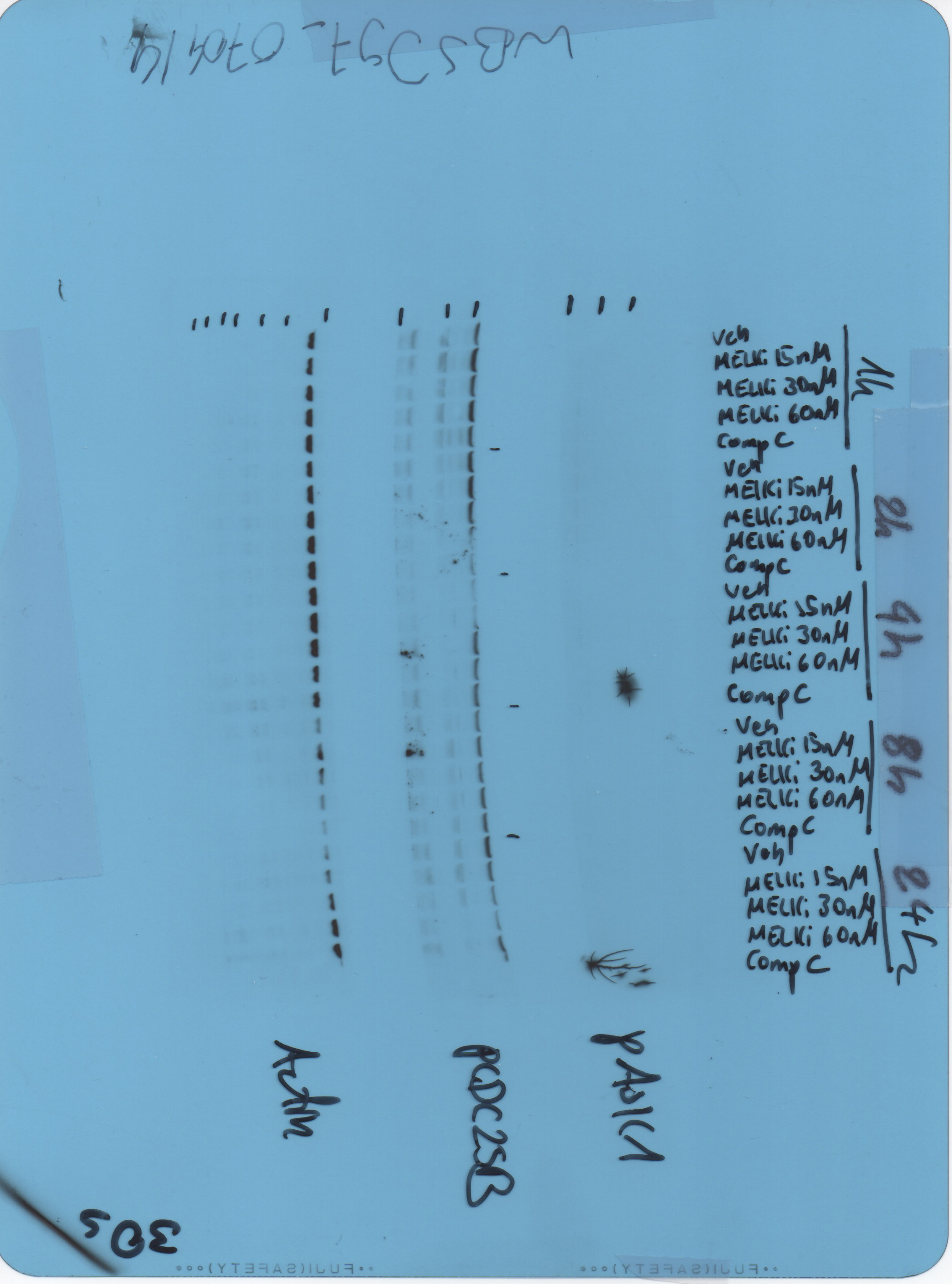
Veh
MELKi 15nM
MELKi 30nM
MELKi 60nM
Comp C
1h
Veh
MELKi 15nM
MELKi 30nM
MELKi 60nM
Comp C
2h
Veh
MELKi 15nM
MELKi 30nM
MELKi 60nM
Comp C
4h
Veh
MELKi 15nM
MELKi 30nM
MELKi 60nM
Comp C
8h
Veh
MELKi 15nM
MELKi 30nM
MELKi 60nM
Comp C
24h

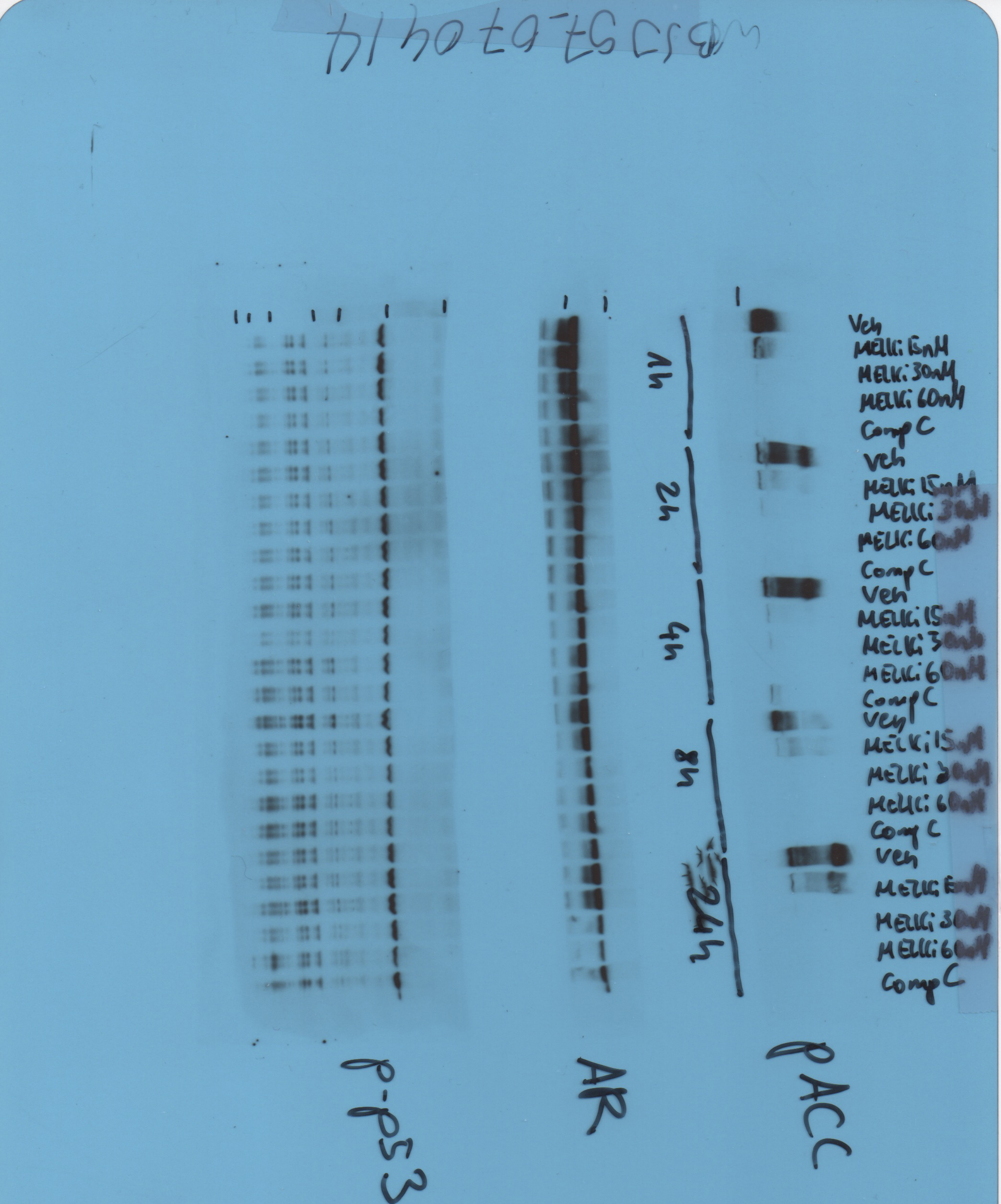
Veh
MELKi 15nM
MELKi 30nM
MELKi 60nM
Comp C
Veh
MELKi 15nM
MELKi 30nM
MELKi 60nM
Comp C
Veh
MELKi 15nM
MELKi 30nM
MELKi 60nM
Comp C
Veh
MELKi 15nM
MELKi 30nM
MELKi 60nM
Comp C
Veh
MELKi 15nM
MELKi 30nM
MELKi 60nM
Comp C
1h
2h
4h
8h
24h
p-p53
AR
pACC

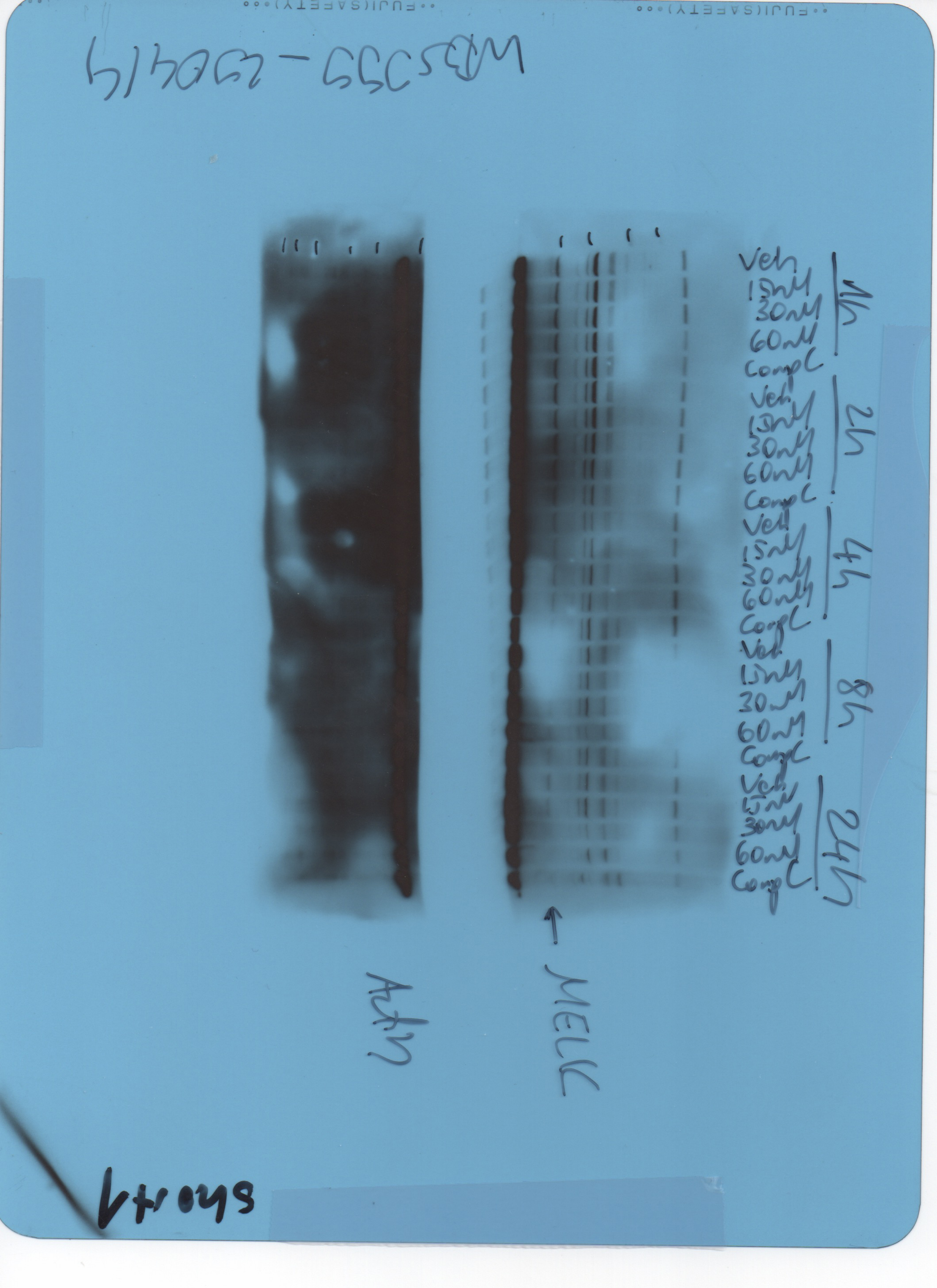
Veh
15nM
30nM
60nM
CompC
1h
2h
4h
8h
24h
Actin
MELK

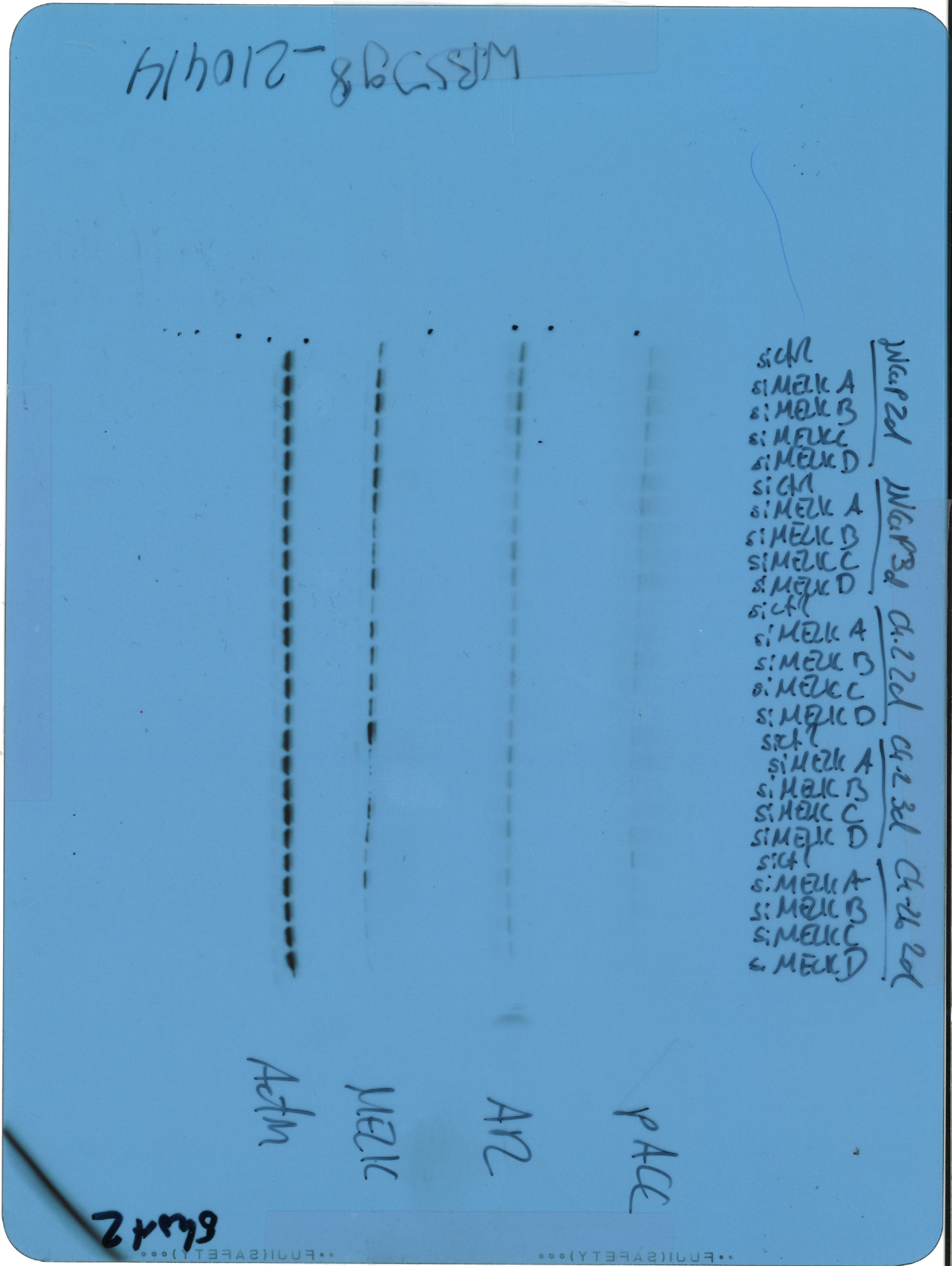

siCtrl
siMELK A
siMELK B
siMELK C
siMELK D
LNCaP 2d
siCtrl
siMELK A
siMELK B
siMELK C
siMELK D
LNCaP 3d
siCtrl
siMELK A
siMELK B
siMELK C
siMELK D
C4-2 2d
siCtrl
siMELK A
siMELK B
siMELK C
siMELK D
C4-2 3d
siCtrl
siMELK A
siMELK B
siMELK C
siMELK D
C4-2b 2d
Actin
MELK
AR

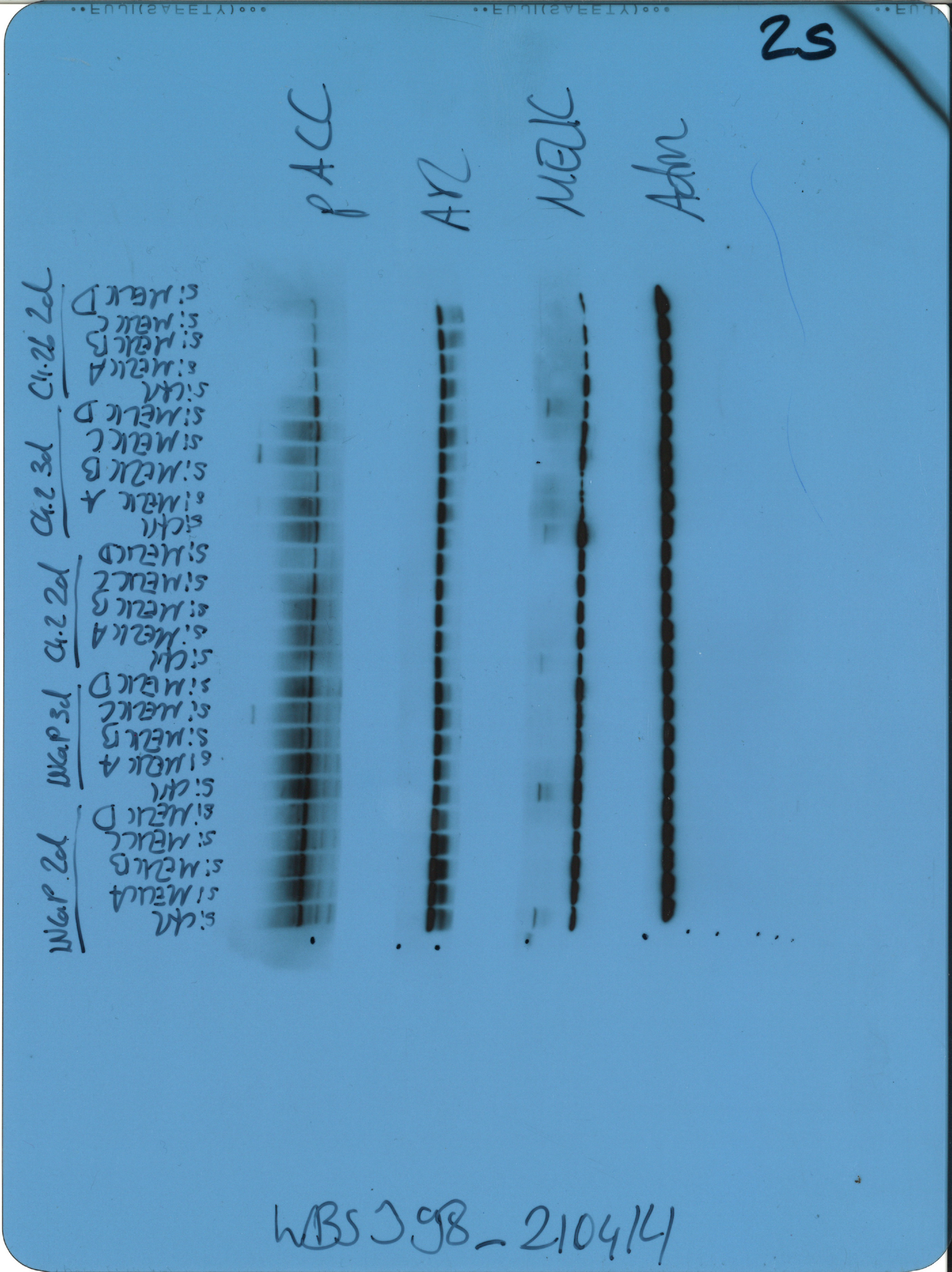
2s
PACC
AR
MELK
Adm
WBSJ98_210414
