# Supplementary material for: Identification of potential therapeutic targets in prostate cancer through a cross‐species approach
Source: EMBO Mol Med. 2018 Feb 5;10(3):e8274. doi: 10.15252/emmm.201708274 (PMC5840539; doi:10.15252/emmm.201708274)
Supplement: Supplementary file 5 — Source Data for Expanded View [file EMMM-10-e8274-s011.zip › SourceDataForFigureEV5/EMM-2017-08274_SourceDataForFigureEV5.pdf]

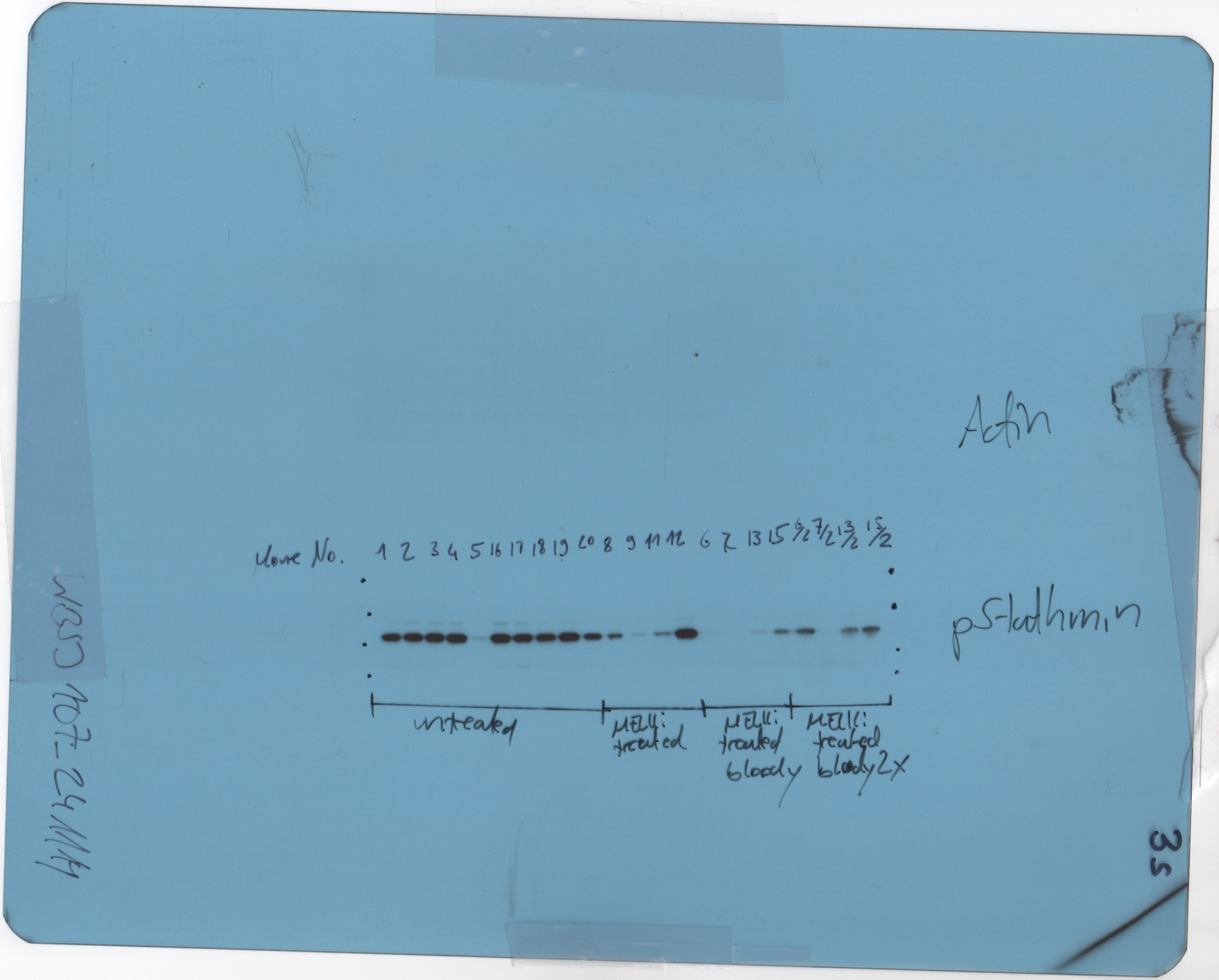
3s
Actin
pStathmin
Mouse No. 1 2 3 4 5 16 17 18 19 20 8 9 11 12 6 7 13 15 6/2 7/2 13/2 15/2
untreated
MELLi: treated
MELLi: treated bloody
MELLi: treated bloody 2x

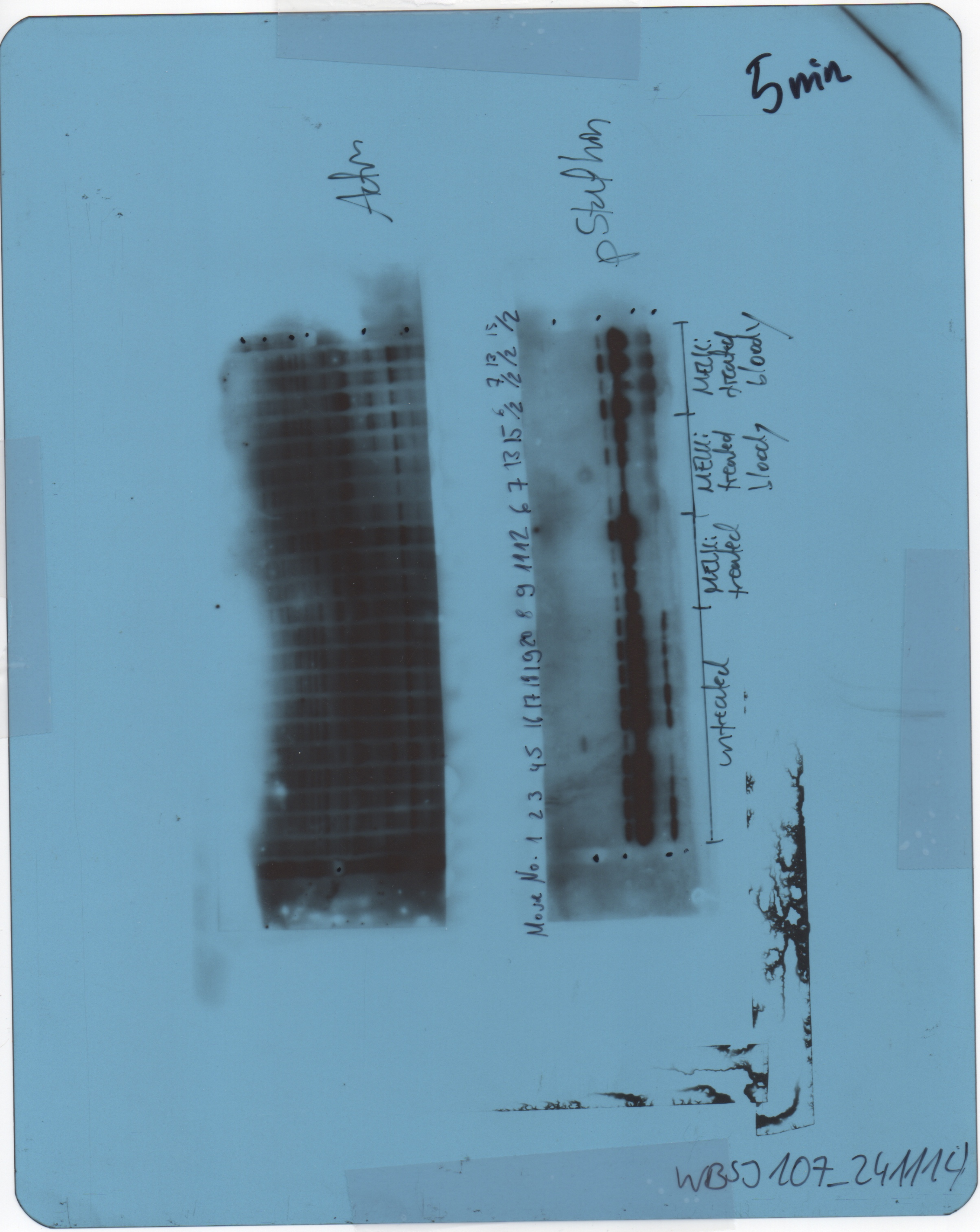
5 min
Actin
Mouse No.
untreated
WBSJ107_241114

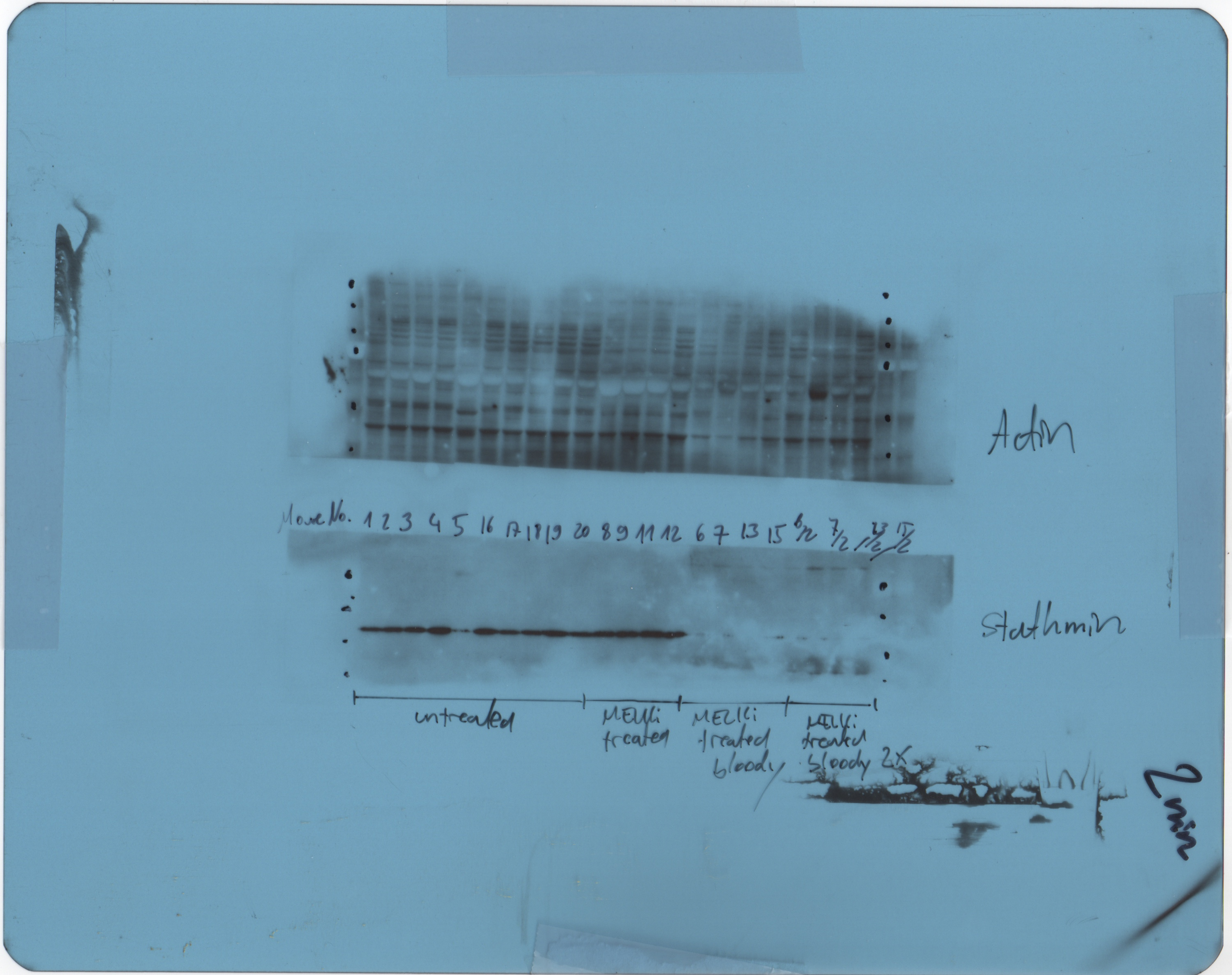
Actin
Mouse No. 1 2 3 4 5 16 17 18 19 20 8 9 11 12 6 7 13 15 6/2 7/2 13/2 15/2
Stathmin
untreated
MEKi treated
MEKi treated bloody
MEKi treated bloody 2X
2 min

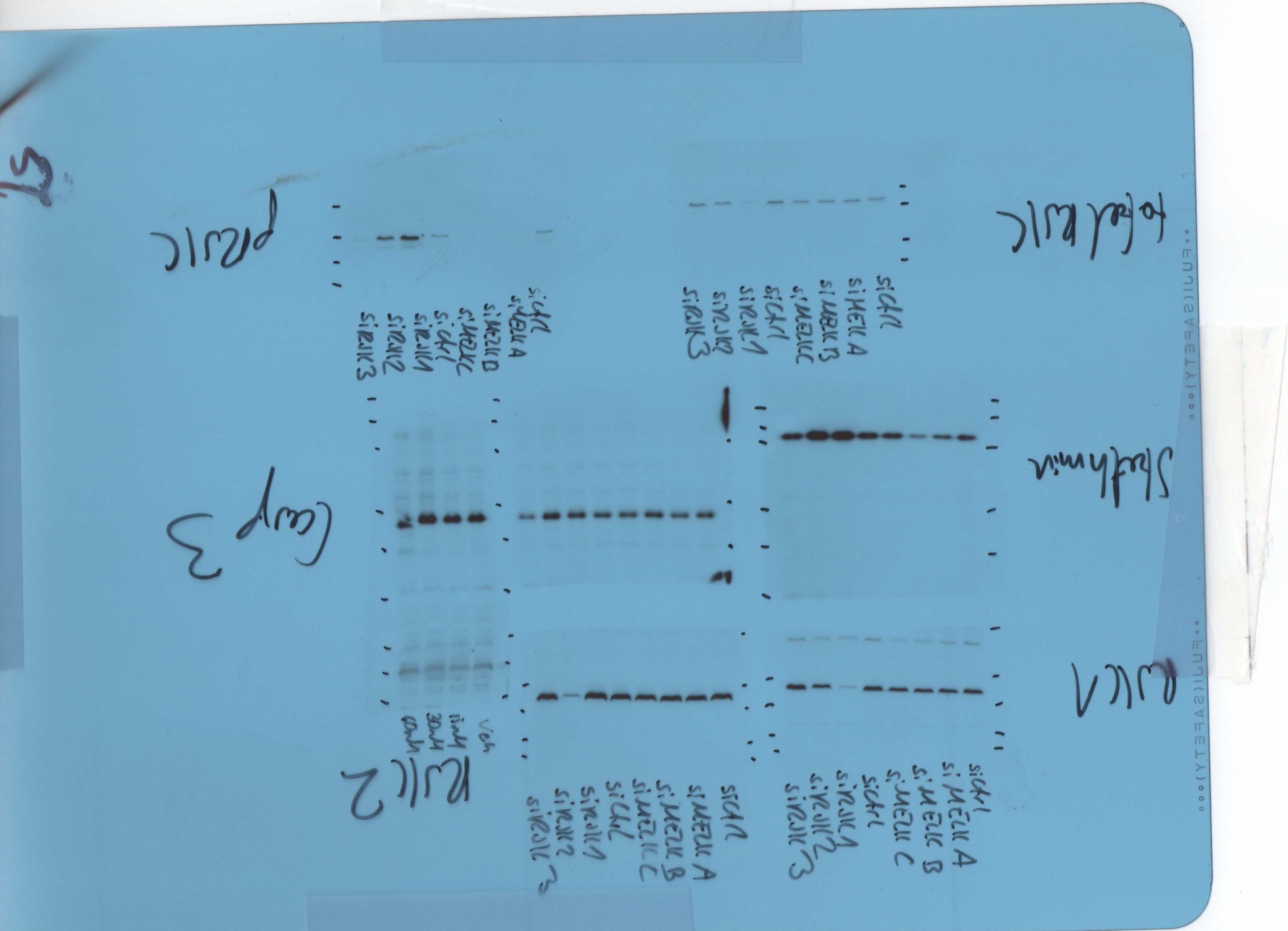

pRSK
total RSK
Casp 3
Stathmin
RSK 2
RSK 1

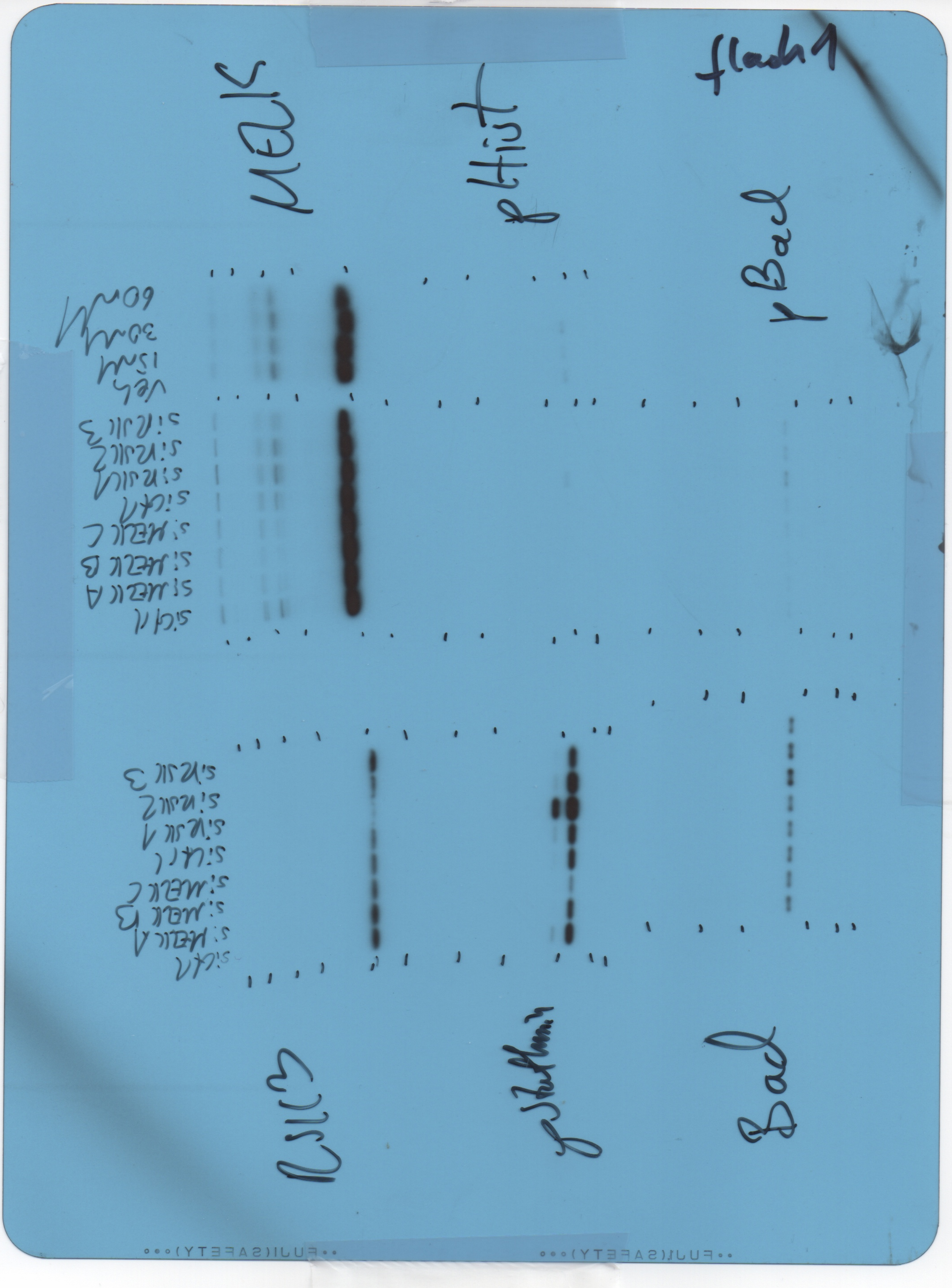
flach1
MELK
pHist
pBad
Bad

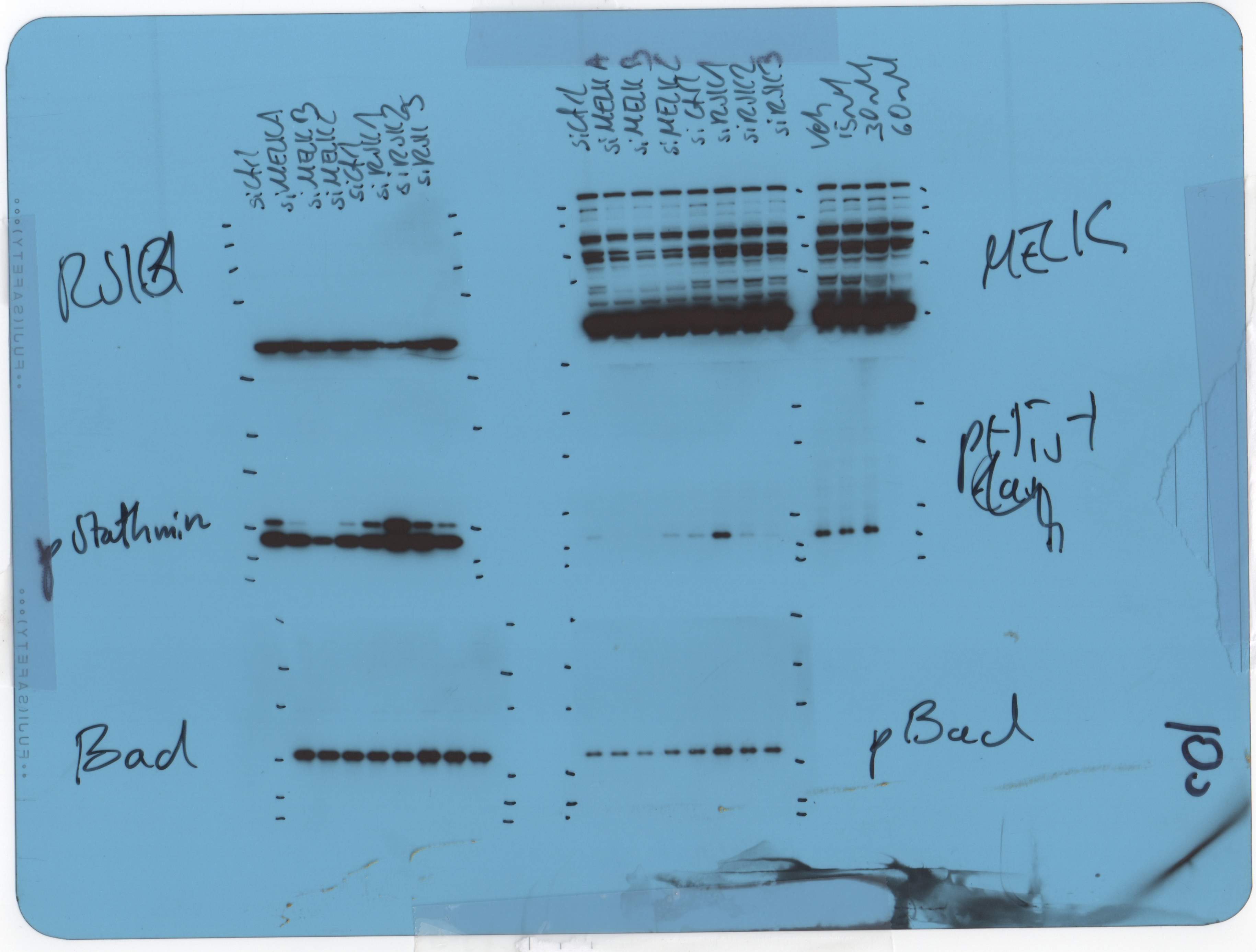
RSK2
pStathmin
Bad
siCtrl
siMELK A
siMELK B
siMELK C
siCtrl
siRSK1
siRSK2
siRSK3
MELK
veh
15nM
30nM
60nM
pBad
Bad

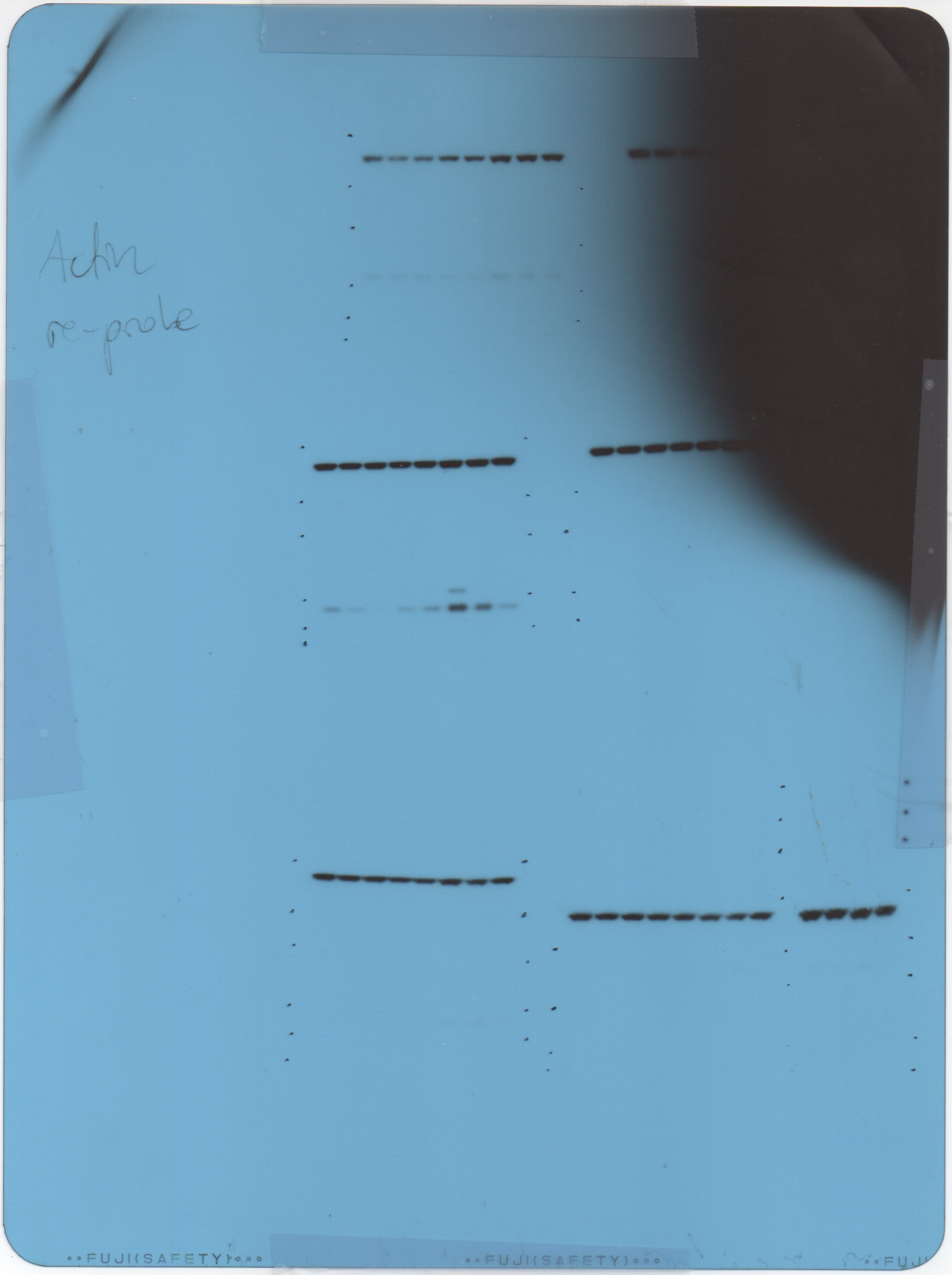
Actin
re-probe
FUJI(SAFETY)
