# Supplementary material for: Identification of potential therapeutic targets in prostate cancer through a cross‐species approach
Source: EMBO Mol Med. 2018 Feb 5;10(3):e8274. doi: 10.15252/emmm.201708274 (PMC5840539; doi:10.15252/emmm.201708274)
Supplement: Supplementary file 12 — Source Data for Figure 7 [file EMMM-10-e8274-s010.zip › EMM-2017-08274_SourceDataForFigure7/EMM-2017-08274_SourceDataForFigure7B-C.pdf]

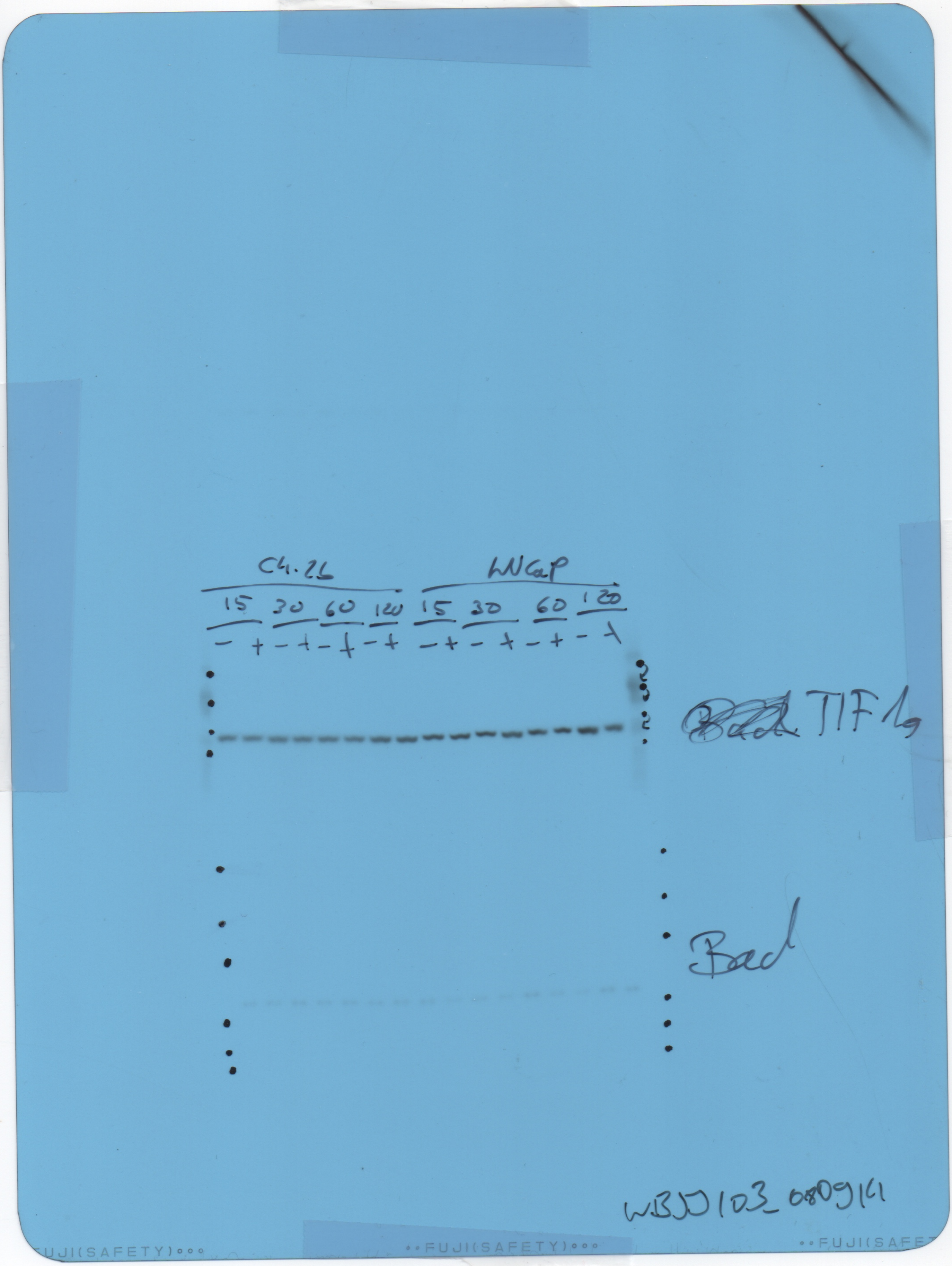
LNCaP
15 30 60 120 15 30 60 120
Bad
WBJJ103_080914
FUJI(SAFETY)

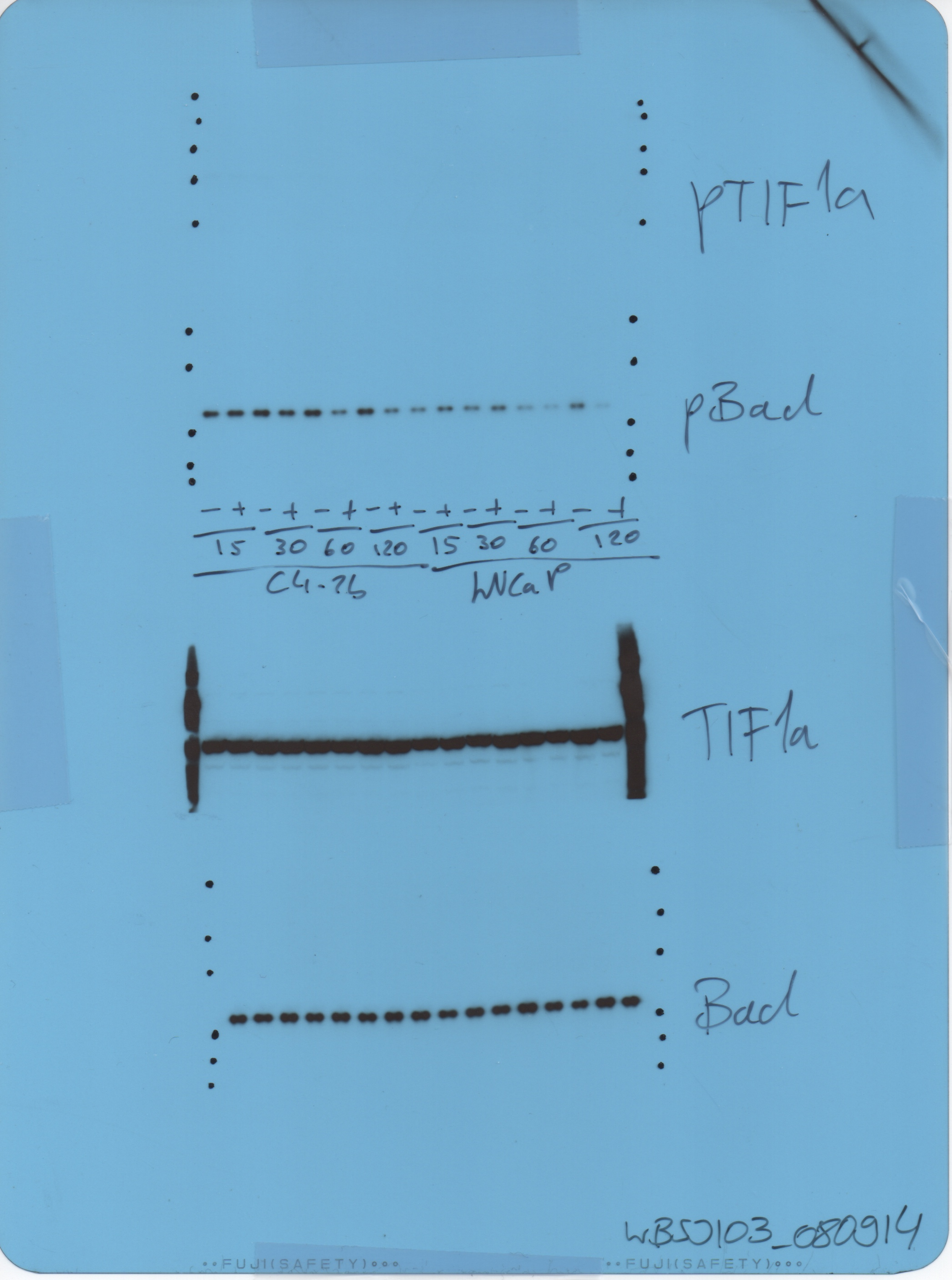
pTIF1a
pBad
- + - + - + - + - + - + - + - +
15 30 60 120 15 30 60 120
C4-2b
LNCaP
TIF1a
Bad
WB5J103_080914
FUJI(SAFETY)
FUJI(SAFETY)

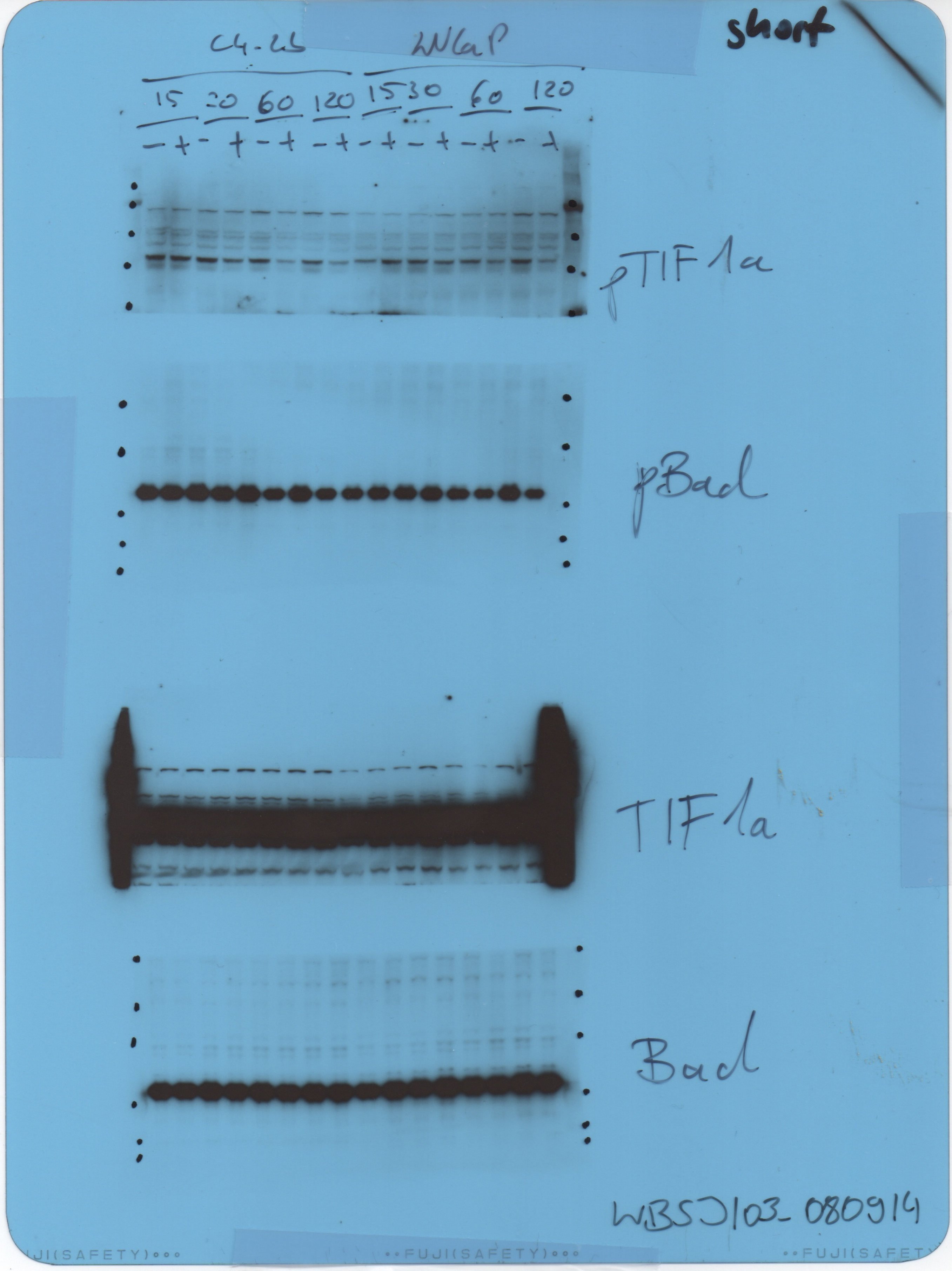
short
C4-2B
LNCaP
15 30 60 120 15 30 60 120
pTIF1a
pBad
TIF1a
Bad
WBSJ103_080914
FUJI(SAFETY)

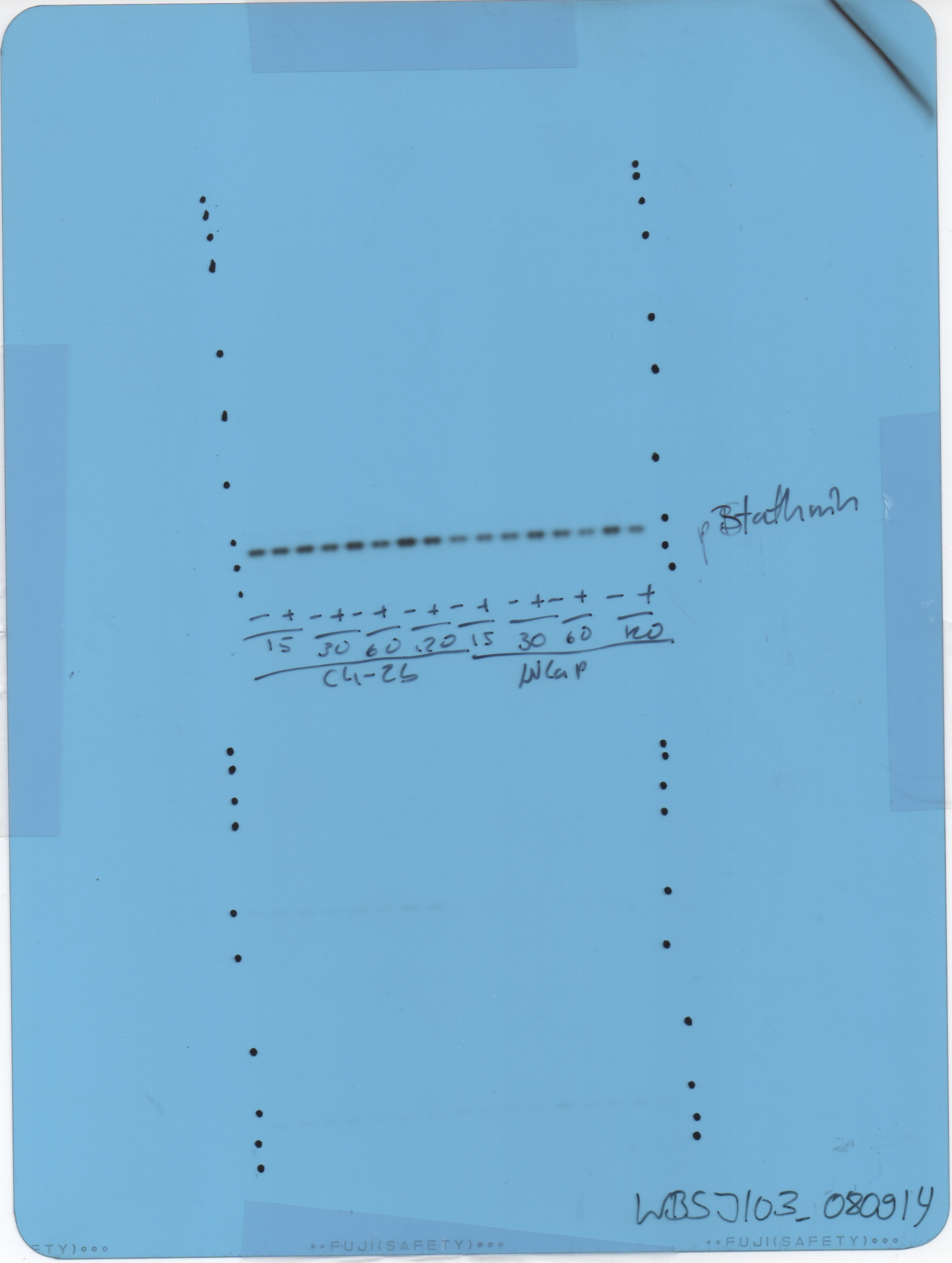
pStathmin
− + − + − + − + − + − + − + − +
15 30 60 120 15 30 60 120
C4-2b
LNCaP
WBSJ103_080914
FUJI(SAFETY)

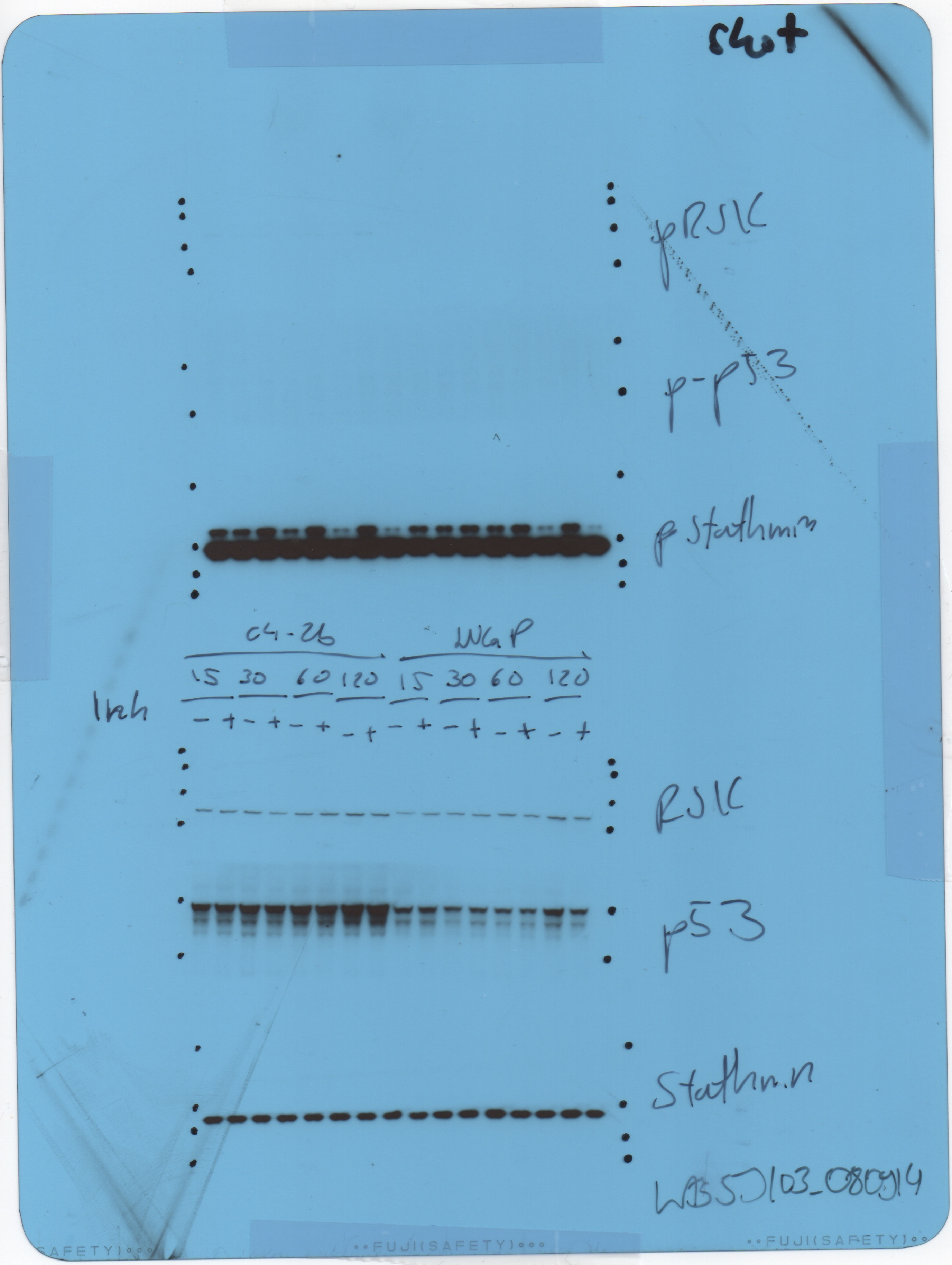
short
pRSK
p-p53
pStathmin
C4-2b
LNCaP
15 30 60 120 15 30 60 120
Inh
- + - + - + - + - + - + - + - +
RSK
p53
Stathmin
FUJI(SAFETY)

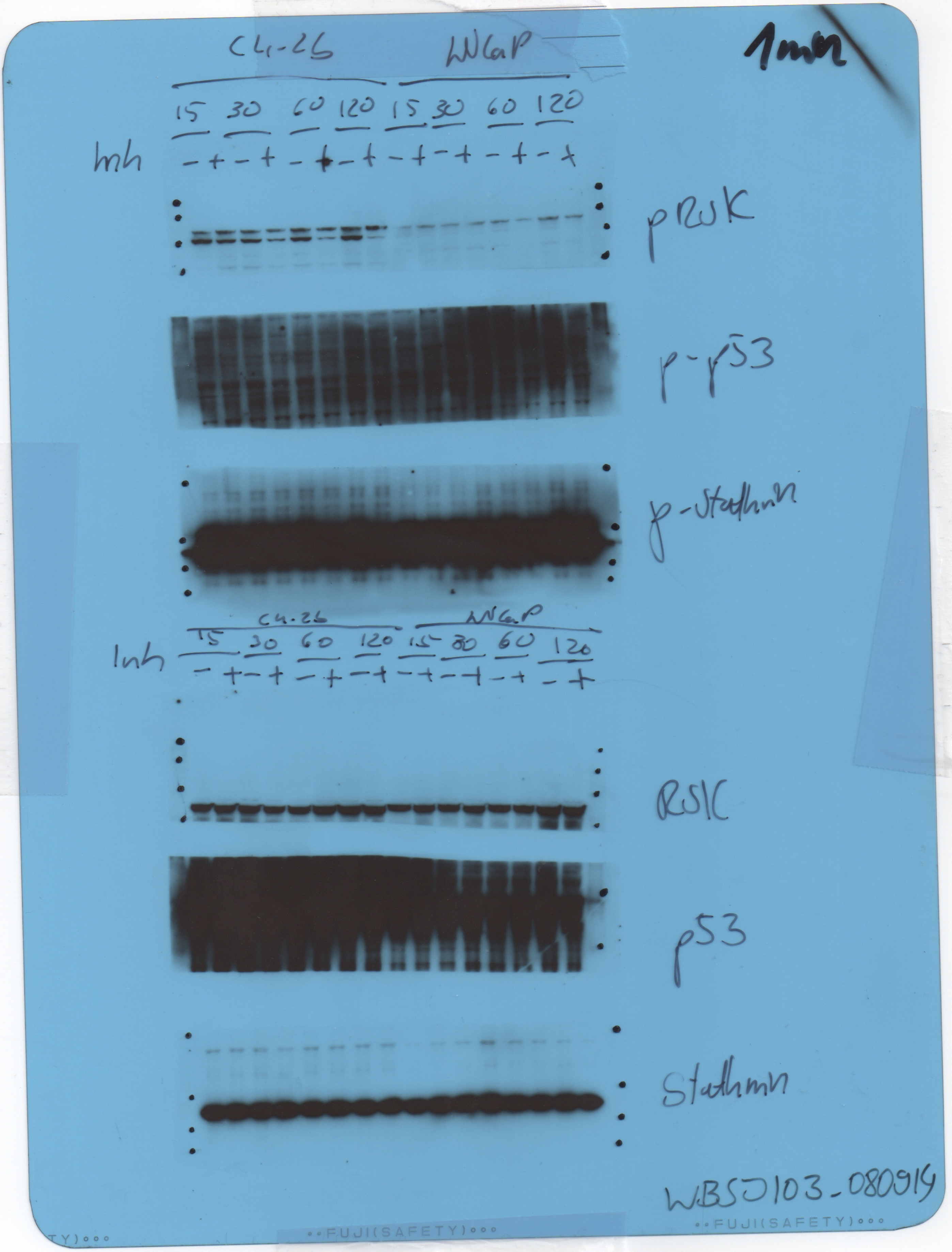
C4-2B
LNCaP
1min
15 30 60 120 15 30 60 120
Inh
pRSK
p-p53
p-Stathmin
RSK
p53
Stathmin
WBSJ103-080914
FUJI(SAFETY)
